# Combination of Group Singular Value Decomposition and eLORETA Identifies Human EEG Networks and Responses to Transcranial Photobiomodulation

**DOI:** 10.3389/fnhum.2022.853909

**Published:** 2022-05-10

**Authors:** Xinlong Wang, Hashini Wanniarachchi, Anqi Wu, Hanli Liu

**Affiliations:** Department of Bioengineering, University of Texas at Arlington, Arlington, TX, United States

**Keywords:** transcranial photobiomodulation, singular value decomposition, eLORETA, default mode network, executive control network, frontal parietal network

## Abstract

Transcranial Photobiomodulation (tPBM) has demonstrated its ability to alter electrophysiological activity in the human brain. However, it is unclear how tPBM modulates brain electroencephalogram (EEG) networks and is related to human cognition. In this study, we recorded 64-channel EEG from 44 healthy humans before, during, and after 8-min, right-forehead, 1,064-nm tPBM or sham stimulation with an irradiance of 257 mW/cm^2^. In data processing, a novel methodology by combining group singular value decomposition (gSVD) with the exact low-resolution brain electromagnetic tomography (eLORETA) was implemented and performed on the 64-channel noise-free EEG time series. The gSVD+eLORETA algorithm produced 11 gSVD-derived principal components (PCs) projected in the 2D sensor and 3D source domain/space. These 11 PCs took more than 70% weight of the entire EEG signals and were justified as 11 EEG brain networks. Finally, baseline-normalized power changes of each EEG brain network in each EEG frequency band (delta, theta, alpha, beta and gamma) were quantified during the first 4-min, second 4-min, and post tPBM/sham periods, followed by comparisons of frequency-specific power changes between tPBM and sham conditions. Our results showed that tPBM-induced increases in alpha powers occurred at default mode network, executive control network, frontal parietal network and lateral visual network. Moreover, the ability to decompose EEG signals into individual, independent brain networks facilitated to better visualize significant decreases in gamma power by tPBM. Many similarities were found between the cortical locations of SVD-revealed EEG networks and fMRI-identified resting-state networks. This consistency may shed light on mechanistic associations between tPBM-modulated brain networks and improved cognition outcomes.

## Introduction

Transcranial Photobiomodulation (tPBM) is an emerging optical neuromodulation method that uses near-infrared (NIR) light (700–1,070 nm) for non-invasive stimulation of cerebral cellular functions (Eells et al., 2004; Wong-Riley et al., 2005; Lampl et al., 2007; Zivin et al., 2009; Barrett and Gonzalez-Lima, 2013). Several studies have reported that the low-power, high-fluence NIR light emitted from lasers or light-emitting diodes (LEDs) can penetrate the extracranial layers of the human head to reach the cerebral cortex and 3–4 cm within the brain (Jagdeo et al., 2012; Henderson and Morries, 2015; Tedford et al., 2015), enabling beneficial neuromodulation to treat a variety of brain disorders or diseases (Rojas and Gonzalez-Lima, 2011; Hamblin, 2016; Hamblin and Huang, 2019). In particular, two recent clinical publications have reported significant improvement of cognitive activity and sustained beneficial effects in patients with dementia by LED-based tPBM over 4-week (Dougal et al., 2021) and 8-week (Nizamutdinov et al., 2021) longitudinal multi-session treatments, respectively. Another clinical study has also evidenced acute augmentation of human memory in dementia patients by single-session tPBM (Chan et al., 2021). Furthermore, single-session delivery of tPBM with 1,064-nm laser on the forehead of healthy humans has been reported to facilitate acute human cognition enhancement across different groups of participants (Barrett and Gonzalez-Lima, 2013; Rojas and Gonzalez-Lima, 2013; Gonzalez-Lima and Barrett, 2014; Blanco et al., 2015, 2017; Gonzalez-Lima and Auchter, 2015; Hwang et al., 2016; Vargas et al., 2017).

The mechanism of action of tPBM is based on the rationale that NIR light gives rise to photo-oxidation of cytochrome-c-oxidase (CCO), the terminal enzyme in the mitochondrial respiratory chain and the main intracellular light-absorbing enzyme in the near-infrared range (Rojas and Gonzalez-Lima, 2011; Hamblin, 2016; Hamblin and Huang, 2019). The oxidized form of CCO (oxi-CCO) plays a key role in the utilization of neuronal oxygen for energy metabolism (Rojas and Gonzalez-Lima, 2011). This CCO-driven mechanism of PBM was first experimentally evidenced by Wang et al. in the human arm and forehead (Wang et al., 2016, 2017; Wu et al., 2019), demonstrating that tPBM at 1,064 nm can non-invasively stimulate mitochondrial metabolism and hemodynamic oxygenation in tissue vasculature. This set of experimental observations was supported by reproducible results (Pruitt et al., 2020) and another independent human study (Saucedo et al., 2021), all of which provide strong evidence for the well-accepted, CCO-driven mechanism of action for tPBM.

However, research on the mechanism of tPBM-evoked electrophysiological effects in the human brain is on its early stage with only a handful of publications (Berman et al., 2017; Vargas et al., 2017; Zomorrodi et al., 2019; Ghaderi et al., 2021; Spera et al., 2021), besides ours (Wang et al., 2017, 2019), in the last 4–5 years. Most of these studies have reported alterations of electroencephalography (EEG) powers by tPBM compared to sham stimulation. In particular, our group recently observed that tPBM enabled to neuromodulate the eyes-closed, task-free human brain, causing increases of EEG alpha and beta powers with significantly distinct topographies compared to those under sham and thermal stimulations (Wang et al., 2021). However, these approaches would not permit spatial identification of brain networks and/or functional connectivity being activated or photo-modulated by given tPBM. It is of great importance if specific brain networks and/or cortical activations can be recognized and localized. As a result, researchers and clinical scientists would be able to link the tPBM-altered brain networks closely with improved human cognition or performance and thus to better understand the underlying mechanism of electrophysiological effects of tPBM. Accordingly, our goal of this paper was to develop a novel EEG data processing methodology that enabled us to achieve human EEG networks and their responses to tPBM.

To accomplish our goal, we conducted a brief literature review on methodologies used for quantification of EEG brain networks at resting state. While many and diverse publications are found with use of EEG-based brain network analysis, only a few explored 3-dimensional (3D) volumetric EEG connectivity based on volume source localization analysis. For example, Aoki et al. in 2015 utilized exact low resolution brain electromagnetic tomography (eLORETA) with independent component analysis (ICA) to resolve EEG resting state functional networks and the respective activities in five EEG frequency bands (Aoki et al., 2015). Custo et al. in 2017 applied k-mean clustering to classify EEG temporal topographies and applied the source localization algorithm to identify respective cortical sources (Custo et al., 2017). Snyder et al. more recently reported brain network functional impairment in stroke patients with respect to healthy participants using 3D volumetric, orthogonalized EEG data analysis (Snyder et al., 2021). The reason of orthogonalizing the EEG time courses was to reduce the effect of volume conduction on connectivity analyses (Snyder et al., 2021). Along the same direction or argument, we explored a novel approach by combining group singular value decomposition (gSVD) and eLORETA to identify human EEG brain networks and responses to tPBM. This is because SVD enables us to separate EEG temporal signal into orthogonal and uncorrelated components, enhancing the independency of EEG networks (2D topographies) with mathematical/scientific rigor.

SVD is a common matrix decomposition algorithm and mainly used to decompose one complex matrix into several orthogonal matrices or components (Bender et al., 2019). While SVD is very useful in many areas of science, engineering, and statistics, it has been extensively applied to solving linear inverse problems, such as for signal processing or imaging processing (Chen et al., 2019; Chowdhury and Dutta, 2019; Ginebreda et al., 2019; Guillemot et al., 2019). Applications of using SVD to decompose multi-channel EEG signals have been reported theoretically and experimentally by previous studies. For example, Shahid et al. applied SVD on EEG signals to identify epileptic seizures (Shahid et al., 2014); Haddad et al. proved the feasibility of using SVD to resolve EEG independent networks (Haddad and Najafizadeh, 2015); Jonmohamadi et al. reported the fused component using EEG and fMRI data with the help of SVD (Jonmohamadi et al., 2019). To the best of our knowledge, no study has examined SVD as an analytical tool to resolve EEG networks and/or quantify tPBM-induced stimulation effects on the respective networks.

Thus, in this study, we hypothesized that group SVD (gSVD) in conjunction with eLORETA enabled to identify (a) human EEG networks on the 2D sensor and 3D source space and (b) their responses to the 1,064-nm tPBM on the right forehead of healthy humans. To test/support our hypothesis, we conducted tPBM and sham experiments concurrently with 64-channel EEG recordings before, during, and after the tPBM/sham stimulation on 44 healthy human participants (Wang et al., 2016, 2017). After implementing the gSVD approach (Harner, 1990; Bai et al., 2019), we were able to recognize or characterize 11 most-weighted, two-dimensional (2D) principal components (PCs) from the gSVD outputs and considered them as dominant EEG brain networks based on minimal temporal correlations among any of them. By performing eLORETA (Jatoi et al., 2014; Imperatori et al., 2015; Ikeda et al., 2019) on these 2D topographies of gSVD-derived brain networks, we further achieved three-dimensional (3D) cortical source locations for each network. Furthermore, the tPBM and/or sham induced power changes on the temporal dynamics of each EEG brain network were quantified in each EEG frequency band (delta, theta, alpha, beta, and gamma). By conducting pair-wise non-parametric statistic permutation comparisons of power changes at each frequency band between tPBM vs. sham conditions, we pinpointed several tPBM-modulated EEG brain networks at alpha and gamma bands, which were consistent with MRI-based brain networks and also highly associated with human cognition and behavior.

## Materials and Methods

### Participants

With the same inclusion and exclusion criteria as those in our previous studies (Wang et al., 2016, 2018, 2021), a total of 49 healthy human subjects were recruited (19 females of 27.4 ± 6.1 years of age; 30 males of 28.7 ± 4.7 years of age) from the local community of the University of Texas at Arlington for participating this study. The experimental protocol was approved by the institutional review board (IRB) at the University of Texas at Arlington and complied with all applicable federal and NIH guidelines. A written informed consent for the sham-controlled tPBM experiment was signed by each participant before each experiment. Four subjects were removed for data analysis because of being asleep during the experiments. Furthermore, one more subject was removed during the data analysis phase, because the power values of 11 SVD components were out of 4 standard deviations of the group mean. Thus, this study analyzed the EEG data sets from a total of 44 human subjects.

### Instrumentation and Experimental Protocol

**FIGURE 1:**
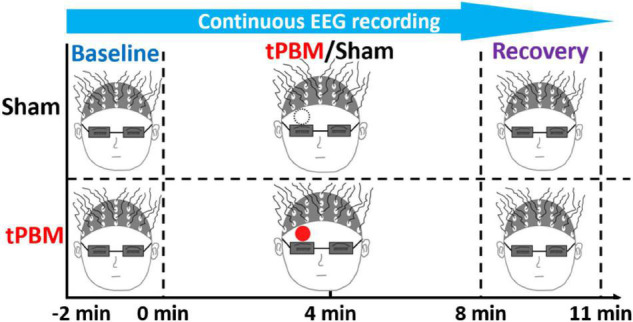
Experimental protocol with a 2-min baseline, 8-min tPBM/sham, and a 3-min recovery period. The solid red and black-dashed open circles mark the spatial sites of tPBM and sham delivery, respectively, on the subject’s forehead. All participants and the experimental operator were required to wear a pair of googles for eye protection.

The sham-controlled experimental protocol was reported earlier (Wang et al., 2021) but briefly shown in Figure 1. Each tPBM or sham experiment took a total of 13 min, ranging from −2 min to 11 min, with a 2-min baseline, an 8-min tPBM/sham, and a 3-min recovery period. During the entire experiment, each participant was required to close his or her eyes but stayed awake. The 2-min baseline recording was designed as a reference for EEG signal normalization to minimize the biological variation of EEG power among individuals. tPBM or sham was delivered on the right forehead above the eyebrow and below the hairline. 64-channel EEG recording was concurrently taken throughout the entire experiment for both sham and tPBM sessions. The sequences of active and sham experiments were randomly assigned; the time interval between the two experiments was at least 3 days to minimize post-stimulation residuals/effects. The participants had no information about sham or true tPBM stimulations until they completed both experiments.

To conduct tPBM, we employed the same type of 1,064-nm laser (Model CG-5000, Cell Gen Therapeutics LLC, Dallas, TX, United States) as that reported in previous studies (Wang et al., 2016, 2019, 2021; Wu et al., 2019). The illumination area of laser for tPBM was 13.6 cm^2^, with a laser power of 3.5 W and a laser aperture diameter of ∼4.16 cm. Thus, the active optical energy (or dose) and energy density (or fluence) delivered to the human forehead by tPBM were 3.5 W × 480 s = 1,680 J and 1,680 J/(13.6 cm^2^) = 123.5 J/cm^2^, respectively. The active power density (irradiance) was to be 3.5 W/(13.6 cm^2^) = 257.4 mW/cm^2^. On the other hands, the power used for sham was set to be 0.1 W. Furthermore, during the actual sham experiment, the laser aperture was further covered by a black cap to avoid any leaky tPBM light delivered to each subject’s head. Therefore, the true light delivery in the sham experiment was 0 mW/cm^2^. The correct power densities for both experiments were confirmed by a sensitive optical power meter (Model 843-R, Newport Corporation, 8 East Forge Parkway, Franklin, MA 02038, United States). Since the laser beam was well collimated, there was negligible difference between the illumination power at the laser aperture vs. that on the human forehead. The consistency of power density 0 vs. 2 cm away from the laser aperture was also confirmed by the power meter (Wang et al., 2021).

A 64-electrode 10-10 EEG system (Biosemi Inc., Barcelona, Spain) was employed to non-invasively record EEG readings for the whole duration of each experiment. Two separate electrodes, Common Mode Sense (CMS) and Driven Right Leg (DRL), were used as the “ground” of the recording system. The sampling rate of EEG recording was 512 Hz for 22 subjects and 256 Hz for the other 22 subjects, respectively, because of some research topics beyond the scope of this paper. All the EEG data collected with the 512-Hz sampling rate were down-sampled to the 256-Hz rate during data pre-processing by EEGLAB (Delorme and Makeig, 2004; Asadi et al., 2020). The highest EEG frequency to be analyzed in this study was 70 Hz, about 1/4 of the 256-Hz sampling frequency. Based on Nyquist’s theorem, our down-sampling process would not alter the measures of frequency powers (Cohen, 2014, 2021); more justification was given in (Wang et al., 2021).

### Data Analysis

**FIGURE 2:**
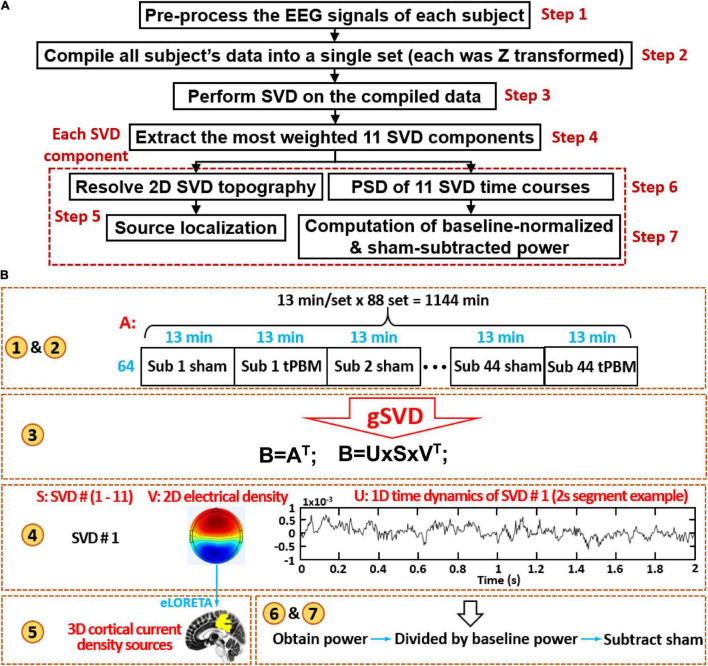
**(A)** A flow chart of seven steps for EEG data processing; **(B)** a corresponding diagram to graphically explain each data processing step in detail. Note that in step 4 of **(B)**, the time course shows only 2-s data as a demo of time series in U matrix for one SVD component. The number in yellow circles matches the steps given in the flowchart **(A)**.

Figures 2A,B display a flowchart and a corresponding diagram, respectively, to describe the seven steps of data processing, including (1) preprocessing for EEG noise removal, (2) concatenation of all subjects’ clean EEG time series (including EEG signal before, during and after tPBM/sham), (3) performance of gSVD, (4) selection of 11 most-weighted principal components (PCs) from the gSVD analysis, (5) cortical source localization of each gSVD component, and (6)--(7) computation of baseline-normalized, sham-subtracted powers for all the gSVD components and at five EEG oscillation frequency bands. All the signal processing operations were carried out using two free-access software packages, EEGLAB (on the platform of MATLAB) and eLORETA.^1^ Specific analysis procedures are described in detail in the following sub-sections.

#### Preprocessing (Step 1)

Data preprocessing was performed on the raw, 64-electrode EEG data using EEGLAB. Because of two sampling rates (512 and 256 Hz) used in the experiments, we first down-sampled the EEG time series with the 512-Hz sampling rate to the 256-Hz time series by a native EEGLAB function “downsample” (Delorme and Makeig, 2004). EEG signal of each electrode was re-referenced to the average of all 64 electrodes. Then, all sets of EEG time series were bandpass filtered between 1 and 70 Hz, followed by a notch filter to remove 60-Hz power line noise. Next, robust principal component analysis (rPCA) (Candès et al., 2011) was applied for effective removal of common artefacts, such as head motions, saccades, and jaw clenches, from our EEG data (Turnip, 2014). Finally, independent component analysis (ICA) (Iriarte et al., 2003; Delorme and Makeig, 2004; Ramkumar et al., 2012) was performed to confirm artifact-free (both temporal and spatial) component patterns/features. Due to the eyes-closed recording condition and the robust performance of rPCA, we did not observe any artifact-caused patterns after ICA for all the participants, and thus no component was further removed before performing further analysis for each participant.

#### Group Singular Value Decomposition (Steps 2–3)

The concept of group SVD in this study can be viewed in analogy to the spatial group ICA that has been widely applied in the field of fMRI (Calhoun et al., 2009; Li et al., 2012). As shown in Figure 2B, after preprocessing, the artifact-free EEG time series matrixes (1–70 Hz bandpass filtered) from all 44 subjects were concatenated into one single matrix, including the 64-channel EEG data from both tPBM and sham sessions of all 44 subjects. Previous studies suggested that there are intrinsic cooperation or interconnection between slow and fast EEG rhythms in mediating brain networks, so the whole frequency band of EEG should be considered together rather than separate it into individual EEG bands (i.e., delta, theta, alpha, beta, and gamma) when doing mathematical operations (Mantini et al., 2007). Next, gSVD algorithm was conducted on the concatenated matrices to resolve common PCs across all the tPBM and sham data sets from all the subjects using the native MATLAB function “svd” with the “economy-size decomposition” setting, which removes extra rows or columns of zeros in S and U, where S and U are explained in Eq. (1). To minimize subjects’ biological variation, individual standardization was performed for each subject’s data before being grouped for concatenation. Specifically, each subject’s EEG time series of each channel was first subtracted by its respective temporal mean, followed by further being divided by its temporal standard deviation. This operation is equivalent to a statistical Z standardization that could avoid non-uniform weight or bias in the SVD calculation from individuals who might have a larger oscillation power, which could bias the common PC computation.

The mathematical expression of “svd” function is shown in Eq. 1:


(1) $$B=U\times S\times V^{T}$$

where B is the transposed matrix of A (i.e., *B* = *A^T^*), and A is the concatenated EEG matrix, U denotes the corresponding vectors of time dynamics for all the 64 vector components; V (64 × 64) denotes the 64 vector components of 2D relative electrical potential (rEP) topographies without a unit (because of the Z transformation on every single subject’s EEG data before gSVD); the diagonal elements of the square S matrix contains 64 singular values of B, indicating the weight of each component decomposed from B. Therefore, as the output of gSVD, we obtained 64 gSVD components as the major PCs, their corresponding 1D time series, and their respective weights over the original EEG signal. This process is graphically shown as steps 2 and 3 in Figure 2B.

#### Extraction of 11 Group Singular Value Decomposition Components (Step 4)

Based on the results obtained in Step 3, we extracted 11 most-weighted gSVD components, presented them in 2D topography in the sensor space, and projected them in 3D cortical source space using eLORETA (details to be given in Step 5). The corresponding 1D time series for each component was segmented into 2-min baseline, 0–4 min tPBM/sham stimulation, 4–8 min tPBM/sham stimulation, and recovery for each participant and for each stimulation type, i.e., tPBM and sham. The reason for making two of 4-min time segments was based on our previous observations that tPBM induced gradual and significant effects only a few minutes after the stimulation onset, and that 4-min temporal segments proved to exhibit meaningful effects of tPBM (Urquhart et al., 2020; Wang et al., 2021) (details to be given in Step 6–7).

#### Source Localization by eLORETA (Step 5)

eLORETA is a free academic software package (see text footnote 1), which converts the 2D scalp distribution of electric potential into cortical 3D distribution of current density using a total of 6,239 voxels at 5-mm spatial resolution to localize electric activity in the human cortex. It offers a weighted least-square based inverse solution with zero localization error under ideal conditions (Pascual-Marqui et al., 2011). In this study, eLORETA was applied on the 2D electric potential distribution (sensor space) of SVD components to localize respective cortical sources (source space). In the operation of eLORETA, the Montreal Neurological Institute (MNI) coordinates of the 64-channel international 10-10 system were employed, and the regularization parameter for generation of the transformation matrix was set to be 1 by default. The above procedures produced 2D (sagittal, coronal, and axial) views as well as 3D cortical maps or representations for each SVD component.

#### Power Spectral Density of Time Course of Each Group Singular Value Decomposition Component (Step 6)

**FIGURE 3:**
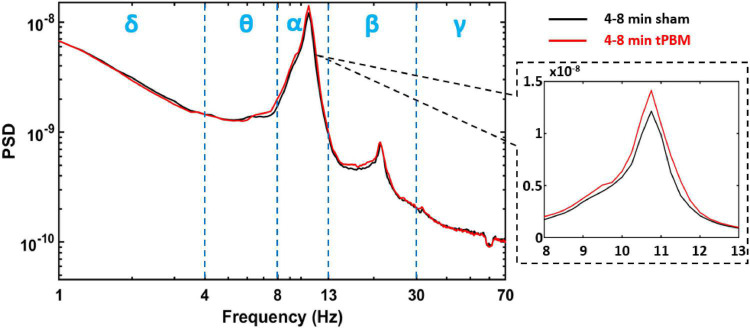
One example of the group-averaged PSD (in log scale for both *x* and *y* axis) for SVD component one, SVD #1, during 4–8 min tPBM (red) and sham (black), respectively. Blue vertical dashed lines separate the five EEG frequency bands, namely, delta (δ: 1–4 Hz), theta (θ: 4–8 Hz), alpha (α: 8–13 Hz), beta (β:13–30 Hz), and gamma (γ: 30–70 Hz) bands. The dashed black box provides a zoom-in view of the PSD in alpha band (in linear scale for both *x* and *y* axis).

For each subject, power spectral analysis was conducted on the time course for each SVD component, respectively. This process resulted in one power spectral density (PSD) curve per component per subject during each temporal segment that we selected (e.g., 2-min baseline, 0–4 min tPBM/sham, 4–8 min tPBM/sham, and 3-min recovery). As an example, Figure 3 demonstrates group-level PSD curves of SVD #1 during the 4–8 min of tPBM (Red) and sham (black). Blue vertical dashed lines separate the five EEG frequency bands into spectral ranges, in which each subject’s spectral power was calculated from its own PSD curve. Note that the zoomed view on the alpha peak of PSD graphically illustrates a potential increase in alpha power compared to that under sham. To quantify power changes in all SVD components statistically, the following processes in Step 7 were conducted.

#### Computation of Baseline-Normalized, Sham-Subtracted Power Changes Induced by Transcranial Photobiomodulation (Step 7)

To compute the absolute tPBM-induced, frequency-specific EEG power changes of each SVD component, we obtained a spectral power by multiplying the averaged PSD value over the chosen spectral band with the corresponding bandwidth for each subject. This operation was repeated for all four time periods (i.e., baseline, 0–4 min tPBM/sham, 4–8 min tPBM/sham, and recovery). Next, baseline normalization was performed by dividing the frequency-specific power during and after tPBM/sham period by its own baseline power. These processes were repeated for all 11 SVD components, for all the subjects, in all five frequency bands, and for all the three periods (0–4 min, 4–8 min, and recovery) for both tPBM and sham conditions, as expressed mathematically in Eqs. (2) and (3), respectively. In this way, a baseline-normalized and sham-subtraction index, ΔnP, was produced per frequency band (*i*) per gSVD component (*j*) per subject (*m*) per time segment (*t*) by subtracting $n\,P_{i}^{j}\,\left(m,t,s\,h\,a\,m\right)$ from $n\,P_{i}^{j}\,\left(m,t,t\,P\,B\,M\right)$, as shown in eq. 4. Indeed, baseline-normalized and sham-subtracted powers reflect the absolute percent changes in power induced by tPBM. As a result, we observed high consistency of PSD curves between the two baselines of tPBM and sham treatment conditions retained in all 11 SVD components, as shown in Supplementary Figure 1. This set of results demonstrated minimal difference in PSD baselines and justified for baseline-normalized comparisons of power changes between tPBM and sham.


(2) $$n\,P_{i}^{j}\,\left(m,t,t\,P\,B\,M\right)=\frac{P_{i}^{j}\,\left(m,t,t\,P\,B\,M\right)}{P_{i}^{j}\,\left(m,b\,a\,s\,e\,l\,i\,n\,e,t\,P\,B\,M\right)},$$

(3) $$n\,P_{i}^{j}\,\left(m,t,s\,h\,a\,m\right)=\frac{P_{i}^{j}\,\left(m,t,s\,h\,a\,m\right)}{P_{i}^{j}\,\left(m,b\,a\,s\,e\,l\,i\,n\,e,s\,h\,a\,m\right)},$$

(4) $$\Delta \,n\,P_{i}^{j}\,\left(m,t\right)=n\,P_{i}^{j}\,\left(m,t,t\,P\,B\,M\right)-n\,P_{i}^{j}\,\left(m,t,s\,h\,a\,m\right),$$

where *nP:* baseline-normalized power;

*ΔnP:* baseline-normalized and sham-subtracted power;

*i*: delta, theta, alpha, beta, and gamma bands;

*j*: 1, 2, 3, … 11 for SVD components;

*m*: 1, 2, 3. … 44 for participants;

*t*: three time periods, 0–4 min, 4–8 min, and 3-min recovery.

Finally, one-sample, non-parametric permutation tests (Manly, 2007; Clements et al., 2021; Krol, 2021) were conducted between the ΔnP vs. zero at the significance level of *p* < 0.05 and *p* < 0.01 for each SVD component, *j*, at frequency band, *i*, and during each of three time periods, *t*, over total participants, *m* = 44. This is essentially equivalent to two-sample, pair-wise, non-parametric permutation comparisons between tPBM and sham, giving rise to bar plots (as shown in Supplementary Figure 3 as an example).

## Results

### Extraction of 11 Most-Weighted Components From Group Singular Value Decomposition Algorithm (Results of Step 3)

**FIGURE 4:**
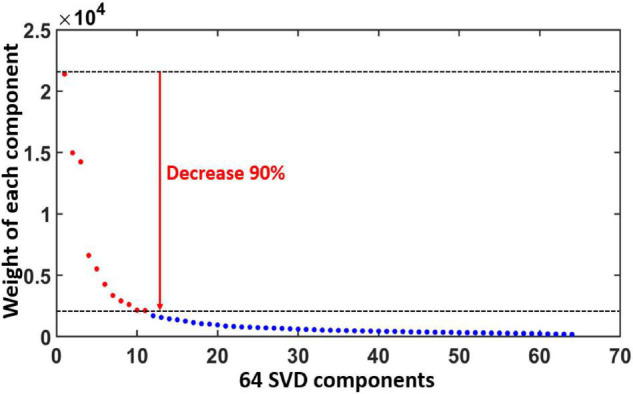
Ranks and weights of all the 64 gSVD components after gSVD process. The *x*-axis indicates the rank number of the 64 components, while the *y*-axis denotes the weight of each component. An exponential decay pattern of weight appears across the 64 components. The 11 most-weighted components, each of which has more than 90% weight of the most weighted component, are marked by red dots and selected for further data processing. The total weight of the 11 selected components (red) takes 70% of the total weight (all the dots).

Results in this section were obtained from Step 3 in data processing. Figure 4 plots the diagonal values of *S* in Eq. 1, demonstrating the ranking of all the 64 PCs/gSVD components based on their weights in EEG signal after gSVD. An exponential decay pattern of weights is shown across the components. In particular, we selected all the components that had less than 90% decay of the first/most-weighted component (=21,500). Thus, all the components with weight factors smaller or lower than 2,150 were excluded, giving rise to 11 dominant components (marked as red color in Figure 4) for further data analyses. Each of these gSVD components can be represented by a 2D topography. The total EEG signals represented by these 11 components accounted for 70% of the entire measured EEG signals (i.e., the area under the curve of all the red regions/dots over that of all the 64 dots in Figure 4).

### Justification of Group Singular Value Decomposition-Derived Independent Brain Networks (Results of Step 4)

**FIGURE 5:**
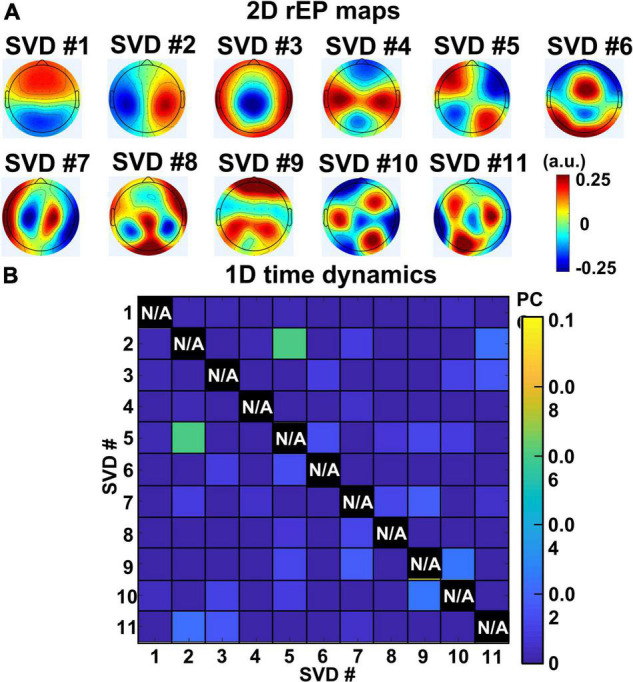
**(A)** 2D topographies of relative electrical potential (rEP) at sensor space for the 11 extracted gSVD components. **(B)** PCC values of temporal dynamics between each pair of gSVD-derived component across 11-min measurement time. It shows that the 11 components have minimally time-correlated values (PCC < 0.07). Note that self-correlation for each component is meaningless and thus marked with N/A (i.e., not applicable).

Results in this section were achieved from Step 4 in data processing. Figure 5A shows 2D topographies of relative electrical potential (rEP) at sensor space for the 11 extracted gSVD components. Clear spatial distinction of rEP was observed across the 11 topographies, indicating the uniqueness and independency of neural activities associated with each SVD component. Moreover, these 2D spatial patterns are highly reproducible regardless of “number of subjects,” “perturbation methods,” and “mental state condition.” Supplementary Figure 2 exhibits the highly repeatable topographical patterns derived by gSVD using “88 data sets of 2-min baseline data,” “44 data sets from tPBM-active measurements through 11-min recordings (i.e., baseline and tPBM and recovery),” “44 data sets from sham measurements through 11-min recordings,” and “random selection of 22 participants” data.

On the other hand, to quantify temporal correlations among all 11 gSVD components, Pearson Correlation Coefficients (PCC) was performed between each pair of the 13-min temporal dynamics of SVD networks for each subject. The group-averaged PCC values between every pair of the 11 gSVD components are shown in Figure 5B, illustrating negligible PCC values (<0.07) for all pairs of components. It provided strong evidence that the 11 gSVD-derived components were temporally independent. Thus, we considered and claimed these 11 PCs as the “EEG brain networks” under the eyes-closed, task-free condition based on their complete independency in both spatial and temporal features among all 11 networks following the widely recognized definitions of large-scale brain networks (Mantini et al., 2007; Raichle and Snyder, 2007; Tagliazucchi et al., 2012).

### Mapping Neural Activities of EEG Networks in Source Spaces (Results of Step 5)

**FIGURE 6:**
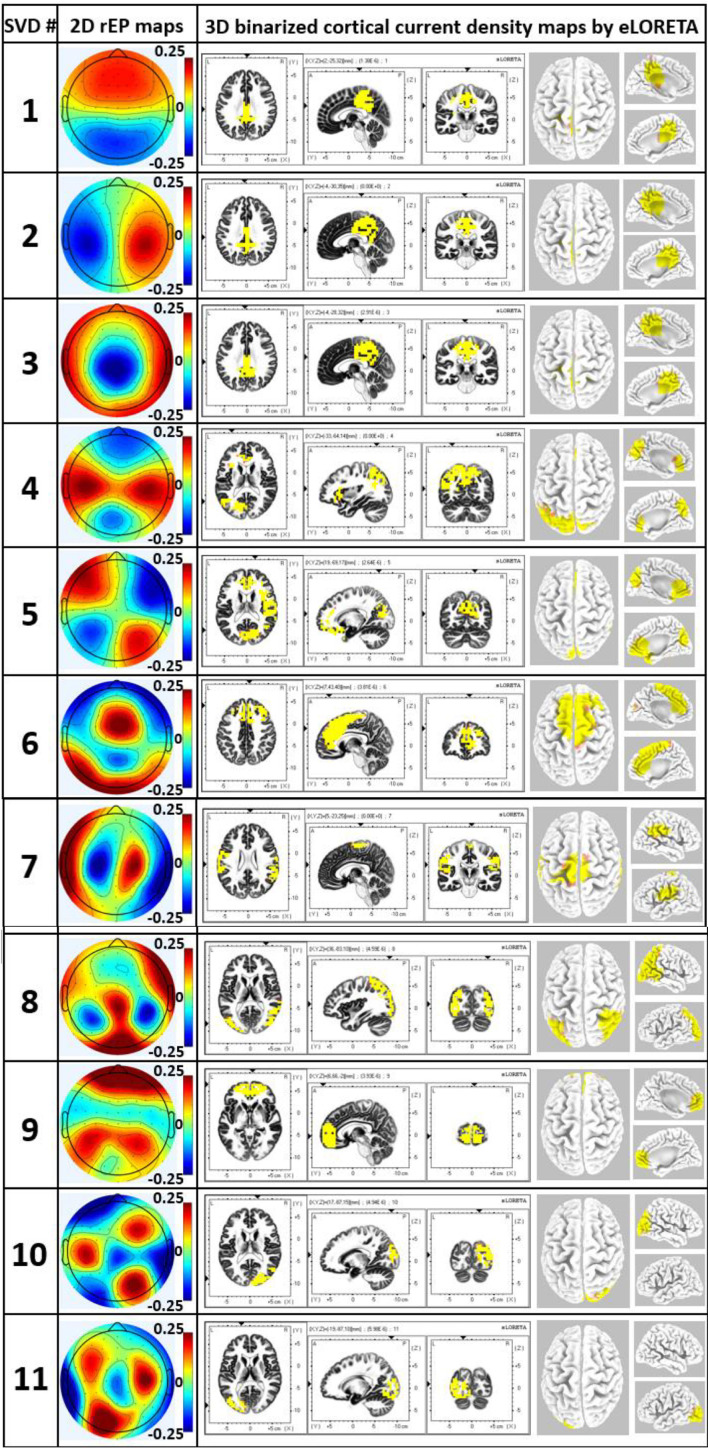
The leftmost column excluding the number column shows 2D rEP maps for 11 EEG brain networks corresponding to 11 SVD components. Accordingly, the middle three columns display axial, sagittal, and coronal views of the current density of neural activity for each EEG network, while the right most column illustrates respective 3D source localizations of cortical current density. Yellow color on the brain models indicates the binarized cortical current density at the associated cortical locations under a threshold of >75% of the maximum neural activity in the network/brain model.

Results in this section were obtained from Step 5 in data processing with the help of eLORETA to compute 3D maps of the cortical/subcortical current density for each EEG brain network, as shown in Figure 6. The leftmost column of Figure 6 shows the 2D topographies of the 11 EEG brain networks. The color bar indicates the relative electrical potential, rEP, of the “dipoles” across the whole scalp without a unit. The middle three columns of Figure 6 display the axial, sagittal, and coronal views of the current density of neural activity. Each panel in the rightmost column exhibits a 3D rendered brain model in top and side views of the left and right hemispheres of each respective EEG brain network. Note that for networks 1, 2, 3, 4, 5, 6, and 9, medial sagittal views of the brain are given to explicitly expose anatomical locations of subcortical sources. On the other hand, for networks 7, 8, 10, and 11, lateral views of the brain are presented to clearly show locations of cortical sources. Yellow color on the rendered brain models indicates the binarized, associated cortical locations under a threshold of >75% of the maximum neural activity (i.e., cortical current density) in the network or 3D brain model. In other words, each voxel in one network was rendered with yellow color if its neural activity was within the top 25 percentile across the 6,239 voxels in the brain model. Note that eLORETA facilitated anatomical and/or structural locations of associated cortical lobes and regions for the 11 EEG networks we identified through gSVD, as listed in Table 1.

**TABLE 1:** Main associated cortical lobes and regions for the 11 networks from eLORETA.

SVD	Associated cerebral lobes	Associated brain regions
# 1	Limbic lobe, parietal lobe	Cingulate gyrus, precuneus
# 2	Limbic lobe, parietal lobe	Cingulate gyrus
# 3	Limbic lobe, parietal lobe	Cingulate gyrus, precuneus
# 4	Left: frontal, parietal lobe	Left: inferior frontal gyrus, inferior parietal lobule
# 5	Right: frontal, parietal, occipital lobe	Right: inferior frontal gyrus, inferior parietal lobule, precuneus
# 6	Medical frontal lobe, limbic lobe	Medial frontal gyrus, anterior cingulate, cingulate gyrus
# 7	Frontal lobe, parietal lobe	Precentral gyrus, postcentral gyrus, inferior parietal lobule
# 8	Occipital lobe, parietal lobe	Middle occipital gyrus, inferior occipital gyrus, superior parietal lobule
# 9	Frontal lobe	Medial frontal gyrus, anterior cingulate
# 10	Right: occipital lobe	Right: middle occipital gyrus, cuneus
# 11	Left: occipital lobe	Left: middle occipital gyrus, inferior occipital gyrus

### Baseline-Normalized, Sham-Subtracted Network Power Changes (Results of Steps 6–7)

**FIGURE 7:**
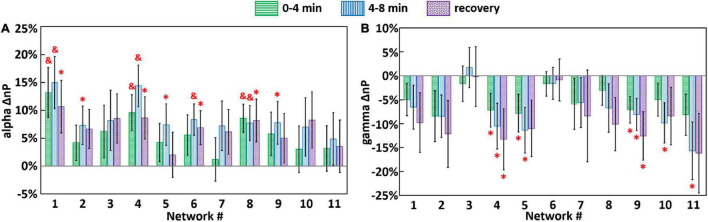
Group-level (*n* = 44) ΔnP (i.e., baseline-normalized, sham-subtracted EEG powers) for each brain network in **(A)** alpha and **(B)** gamma bands, during 0–4 min (green), 4–8 min (blue) tPBM/sham, and 3-min recovery (purple) periods. Error bars represent the standard error of the mean. Significant differences of ΔnP between tPBM vs. sham when pair-wise, two-sample, non-parametric tests between nP values for tPBM and sham (equivalent to one-sample non-parametric test between ΔnP vs. zero) were performed at the significance level of *p* < 0.05 (marked by “*”) and *p* < 0.01 (marked by “&”).

For both tPBM and sham conditions, Eqs. (2) and (3) were utilized to quantify frequency-specific, baseline-normalized network powers during three time periods (0–4 min, 4–8 min, and 3-min recovery) for each of the 11 brain networks. Next, following Eq. (4), the baseline-normalized, sham-subtracted index, ΔnP, was calculated in each frequency band and for each brain network. Figure 7 shows the group-level (*n* = 44) ΔnP in alpha and gamma bands. Specifically, Figure 7A shows that in most of SVD networks across all three time periods, tPBM enhanced alpha powers in networks 1, 4, and 8 significantly across all three time periods, namely, through entire 8-min tPBM and 3-min recovery after removal of sham effect. Moreover, network 6 became significantly augmented during 4–8 min and post tPBM, while alpha powers of networks 2, 5, 9 were significantly boosted only within the 4–8 min stimulation phase. After checking the corresponding cortical locations listed in Table 1, results in Figure 6A revealed that tPBM enabled to neuromodulate EEG alpha powers with a sustained period in numerous cortical and subcortical locations, such as the cingulate gyrus, precuneus, left inferior frontal and parietal lobules, medial frontal gyrus, anterior cingulate, middle and inferior occipital gyrus, and the superior parietal lobule. On the other hand, tPBM did not generate any significant modulation on EEG delta, theta, and beta powers with comparative plots shown in Supplementary Figure 3.

In contrast, as shown in Figure 7B, tPBM significantly decreased EEG gamma powers of networks 4 and 9 throughout all three time periods. Also, network 5 power became significantly reduced during the onset of tPBM and recovered after the stop of tPBM. After close inspection on the brain regions in each SVD network given in Table 1, we suggested that tPBM tended to lower gamma oscillations at the bilateral inferior frontal and parietal lobule, medial frontal gyrus, and anterior cingulate.

## Discussion

Given the previous findings that right-prefrontal tPBM could enhance cognitive functions (Barrett and Gonzalez-Lima, 2013; Rojas and Gonzalez-Lima, 2013; Gonzalez-Lima and Barrett, 2014; Blanco et al., 2015, 2017; Gonzalez-Lima and Auchter, 2015; Hwang et al., 2016; Vargas et al., 2017) and modulate EEG power globally in the human brains (Wang et al., 2017, 2019), we hypothesized that the locally delivered tPBM can neuromodulate the power of frequency-specific EEG brain networks in the task-free human brain, which may result in significant effects on human cognition beneficially. To support or prove our hypothesis, we developed a novel methodology by combining the gSVD algorithm with eLORETA to identify and localize 11 dominant EEG networks presented in both 2D scalp and cortical source space. Several key and/or novel findings are reported in the following sub-sections.

### Large-Scale Neural Activities Presented by Group Singular Value Decomposition-Derived EEG Brain Networks

#### Similarity of Group Singular Value Decomposition-Derived EEG Brain Networks to fMRI-Defined Networks

The application of gSVD in this study enabled the isolation and identification of 11 intrinsic EEG brain networks. In Figure 4, these 11 networks take 70% of the total contribution in the recorded EEG signal. This is in great consistency with respect to the well-known neural physiology that the human brain takes 60–80% of energy in supporting the communication among neurons and functional activity (Raichle and Snyder, 2007). Furthermore, by careful inspection on the active cortical localizations of each EEG network shown in Figure 6 and Table 1, we recognized spatial or cortical co-localizations between the SVD-identified EEG networks and fMRI-recognized networks (Jann et al., 2010; Veer et al., 2010; Aoki et al., 2015; Shen, 2015; Piano et al., 2017; Jonmohamadi et al., 2019). This indicates that, although EEG recorded cerebral electrophysiological oscillations at much higher frequencies with lower spatial resolution than fMRI, SVD-derived EEG brain networks may potentially reflect or share the contribution from the same neural activity with fMRI-defined brain networks. Supplementary Table 1 shows the most associated Brodmann Areas (BAs) of each EEG brain network found in this study and the corresponding fMRI-identified networks covering similar active BAs. For example, gSVD-derived EEG networks # 1–3 are co-located anatomically with the posterior default mode network (pDMN) of fMRI. gSVD-derived EEG networks #4 and #5 share the anatomical sites with the left and right fontal-parietal networks (L- or R-FPN). EEG networks #6 involves the anatomical sites with the executive control networks (ECN). Note that, for some of the fMRI networks, inconsistent names have been used in the literature. Thus, the right-most column of Supplementary Table 1 lists all the names of brain networks used by different publications.

#### Three EEG Networks Sharing Posterior Cortical Sources of Neural Activity

Notably, it is difficult to physiologically understand why the first three EEG brain networks, # 1–3, seemed to be linked to the same single fMRI-identified brain network, namely, parietal DMN. The reason that they were sorted as three individual/separate EEG brain networks is because of the independent features in the spatial distribution of rEP (i.e., having distinct 2D topographies) and in the temporal dynamics (i.e., small PCC values < 0.07), as demonstrated in Figure 5 and Supplementary Figure 4. The reason why they were not resolved in the cortical source domain might be because of the limited spatial resolution that eLORETA is able to achieve for high-spatial source localization.

Compared to fMRI, EEG signals can achieve a much higher temporal resolution with a much higher frequency range of interest. In this study, the frequencies of interest ranged between 1 and 70 Hz, while the frequency span of interest in fMRI is ∼0.1 Hz (Huotari et al., 2019; Yuen et al., 2019). The temporal and spectral differences between the two measurement/image methods may result in different and/or complementary characteristics discovered from the same network. Accordingly, EEG networks #1–3 could be attributed to three independent sources of EEG neural activities/networks that have distinct temporal features but are located around the posterior cingulate gyrus and/or the precuneus regions. Because of much lower sampling frequency of fMRI than EEG, the three spatially adjacent but temporally independent sources may not be temporally resolved by fMRI. In the meantime, due to the lower spatial resolution of EEG, the spatial distinction among the three independent sources could not be visualized/identified as the results of eLORETA analysis.

#### Advantage of Group Singular Value Decomposition to Monitor Neural Activities in Cognition-Sensitive Networks

As can be observed in Figures 4, 6, and Supplementary Table 1, the first 6 most weighted gSVD-derived EEG networks took more than 60% (area under the curve of the first 6 points in Figure 4) of the total EEG signal contributions and co-located with the fMRI-recognized, cognition-sensitive networks, such as DMN, FPN and ECN. This set of observations revealed a potential advantage of the gSVD + eLORETA algorithm that facilitates EEG network-based monitoring of fast neural activities in these cognition-related networks.

The DMN is one of the most dominant and important networks in the human brains (Mohan et al., 2016; Murphy et al., 2018; Sormaz et al., 2018). Our gSVD-derived EEG brain networks found are consistent with this statement. Specifically, four out of the 11 most-weighted EEG networks (i.e., SVD # 1, 2, 3 and 9) were found being associated with the DMN. The weights of these four components (shown in Figure 4) took 66.4 and 47.8% of the top 11 networks and the whole EEG signal (64 components). Thus, due to its dominancy in EEG signal, the gSVD-detected DMN activity are expected to have a high signal-to-noise ratio, offering a potentially rapid and feasible means to extract DMN fluctuations of the human brain from EEG recordings.

Although the DMN is believed active during mind wondering and wakeful rest, recent studies have reported the important role DMN plays during many cognitive activities, such as attention (Dastjerdi et al., 2011), memory encoding (Sormaz et al., 2018), and memory consolidation (Lefebvre and D’Angiulli, 2019). Moreover, DMN is closely related to psychological conditions and mental health of the human brain (Buckner et al., 2008; Akiki et al., 2018). Therefore, the ability to sensitively detect DMN activity by EEG will advance/broaden EEG’s applications as a new neural monitoring tool to complement fMRI with high temporal resolution.

Apart from the DMN, FPN (left and right) and ECN are the next three predominant EEG networks identified by the gSVD algorithm, ranked as # 4–6 and preceded only by the DMN. They took a total of 20.4% weights among the 11 dominant networks. It is known that the FPN plays a key role in human cognition (Jann et al., 2010; Marek and Dosenbach, 2018), including attention and memory during tasks, such as memory encoding and cognitive flexibility (Chan et al., 2008; Kawasaki et al., 2010; Collins and Koechlin, 2012; Diamond, 2013; Nielsen et al., 2017). Likewise, the ECN is vital to human executive control functions during attention and other tasks, such as attentional control, cognitive inhibition, inhibitory control, working memory, planning, reasoning, and problem solving (Janicak, 2002; Chan et al., 2008; Diamond, 2013). Being able to detect neural activities in FPN and ECN would make EEG more practically and broadly useful for rapid, high-temporal-resolution monitoring of cognitive functions in the human brain.

In addition, The significantly modulated alpha and gamma power on DMN, FPN and ECN observed in this study provided a potential mechanistic link or association between tPBM and its impact on improvement of behavioral performances in human memory, attention performance (Jahan et al., 2019), top-down mental processes (Osipova et al., 2008; Voytek et al., 2010) and executive performance (Barrett and Gonzalez-Lima, 2013; Rojas and Gonzalez-Lima, 2013; Gonzalez-Lima and Barrett, 2014; Blanco et al., 2015; Gonzalez-Lima and Auchter, 2015).

### Enhancement of Alpha Power by Transcranial Photobiomodulation in Selected Group Singular Value Decomposition-Derived EEG Brain Networks

Our results in Figure 7A demonstrated that after the self-baseline normalization to reduce biological variations of the brain state, tPBM intervention tended to increase EEG alpha powers in several EEG brain networks, as compared to those under sham condition. Significant modulation effects displayed a network-dependent manner with significantly enhanced alpha power at SVD networks 1 (pDMN), 4 (left-FPN), 6 (ECN), and 8 (lateral visual network; LVN) throughout the during and post-tPBM period while SVD networks 2, 5, 9 were boosted only in the last 4 min of tPBM. According to previous reports by other research groups and ours, alpha power enhancement has been consistently observed as a main effect of tPBM (Wang et al., 2019, 2021; Zomorrodi et al., 2019; Yao et al., 2020). This consistent observation demonstrates the reproducibility of the tPBM effects in the human brains. Also, since alpha wave is one of the essential brain oscillations for cognitive functions (Clements et al., 2021) and neurofeedback (Angelakis et al., 2007), enhanced alpha power may indicate potential benefits to the human cognition. However, in previous studies, the observed enhancements in alpha power were usually reviewed or presented with a large cortical area of the human brain in the EEG topographical format. There was a lack of association between the modulation effects and brain networks to localize specific cortical regions being neuromodulated by tPBM. Thus, it was difficult to explain the previously observed cognition enhancement with direct neuromodulation effects.

The implementation of gSVD enabled the time-spatial decomposition of the 64-electrode EEG signal (Bender et al., 2019), following a similar concept or analogy to the process of principal component analysis (Lagerlund et al., 1997; Wall et al., 2003). In other words, the gSVD-derived components from EEG can be regarded as principal components or dominant networks of the EEG signal. This operation allowed us to visualize and analyze the EEG activities with 64 orthogonal dimensions, followed by dimension reduction using the scale of the singular values (the diagonal values of *S* in Eq. 1, which equals to the root-mean-square amplitude of each spatiotemporal feature (Harner, 1990)) to select the most weighted effects by tPBM.

Furthermore, using eLORETA, the 64-channel electric potential dipoles could be further reconstructed to localize their cortical/sub-cortical sources (Pascual-Marqui et al., 2011). Table 1 revealed that the previously observed global alpha power enhancement originated from cortical neuromodulation effects of the cingulate gyrus, precuneus, bilateral inferior frontal and parietal lobules, medial frontal gyrus, anterior cingulate, middle and inferior occipital gyrus, and the superior parietal lobule. All these cortical regions are responsible for human cognitive functions. For example, the cingulate gyrus is essential for human memory process (Kozlovskiy et al., 2012; Leech and Sharp, 2014); the precuneus is active in memory tasks (Wallentin et al., 2006) and episode memories (Lundstrom et al., 2003); the inferior frontal and parietal lobules are responsible for social cognition, decision making, and attentional performances (Binder and Desai, 2011; Tops and Boksem, 2011; Numssen et al., 2021); the anterior cingulate and the medial frontal gyrus are majorly involved in emotion (Stevens et al., 2011) and decision making (Euston et al., 2012); the occipital gyrus is in charge of perception and object recognition (Grill-Spector et al., 2001); and the superior parietal lobule is essential for the information manipulation during working memory (Koenigs et al., 2009). Therefore, we speculated that the tPBM-induced augments of human cognitive functions (Barrett and Gonzalez-Lima, 2013; Rojas and Gonzalez-Lima, 2013; Gonzalez-Lima and Barrett, 2014; Blanco et al., 2015; Gonzalez-Lima and Auchter, 2015) may result, at least partially, from the enhancement of alpha powers in those cognition-active EEG cortical networks.

### Reduction of Gamma Power by Transcranial Photobiomodulation in Selected Group Singular Value Decomposition-Derived EEG Brain Networks

In addition to the effect on alpha power of EEG networks, significant reduction of gamma power was also observed in this study after exclusion of sham effects. Figure 7B displayed decreases of gamma power in networks 4 (left-FPN) and 9 (DMN) throughout all three time periods. Network 5 (right-FPN) became significantly weaker during the onset of tPBM and recovered after the stop of tPBM compared to the sham stimulation condition.

In our previous study, using the same datasets with a non-decomposition data processing method, the significant decrease of gamma power was not revealed (Wang et al., 2021). This is expected because, in our previous study, the gamma oscillation in EEG signal was grossly being processed with a mixture of all the networks; in this study, the novel application of gSVD was able to decompose 11 principal components or networks from the EEG signal, including respective responses to tPBM. As seen in Figure 7B, among the 11 EEG networks, only five of them showed significant responses. It is expected that a mixture of EEG networks, more than 50% of which had non-significant responses to tPBM, would have much less sensitivity to respond to tPBM. This was why previous analysis did not observe any significant change in gamma power. In turn, the capability of detecting gamma power reduction emphasized another advantage of the gSVD analysis: being able to sense changes in distributed EEG networks induced by different conditions, such as tPBM.

According to Table 1 and Figure 7B, the major cortical regions with modulated gamma oscillation included the bilateral inferior frontal and parietal lobule, the right precuneus and cuneus, the medial frontal gyrus, anterior cingulate, left inferior occipital gyrus, and the bilateral middle occipital gyrus. Additionally, tPBM also modulates the gamma power at the bilateral middle occipital gyrus, which is essential for the mediation of auditory and tactile information (Renier et al., 2010) as well as for the processing of cognitive biases for depression (Teng et al., 2018). Thus, the tPBM-modulated gamma power at the above cortical locations can potentially be used to explain the improved cognitive functions (Barrett and Gonzalez-Lima, 2013; Chaudhari et al., 2021).

To the best of our knowledge, the decrease of gamma power has essential neurophysiological significances. Zhou et al. reported that suppressed alpha power and increased gamma power are related to neural pain (or neuralgia) (Zhou et al., 2018). Tanaka-Koshiyama found that elevated gamma power is associated with learning and memory dysfunction and Schizophrenia (Tanaka-Koshiyama et al., 2020). Fitzgerald et al. reviewed in literature that increased power of gamma wave was a biomarker of depression (Fitzgerald and Watson, 2018). In this study, we observed that tPBM facilitated enhancement of alpha oscillation power and decrease of gamma oscillation power, creating exactly opposite effects to those from the mentioned brain disorders. Thus, the reduction of gamma power in EEG networks by tPBM is another piece of supporting evidence that tPBM may create multiple beneficial and therapeutic effects on the human brain.

### Limitations and Future Work

While our exploratory work revealed several novel findings on tPBM-evoked neuromodulation on human EEG brain networks, we acknowledge several limitations of the current study and point out potential development for future investigations.

First, although many spatial similarities were found between the EEG-derived brain networks and the fMRI-recognized networks in this study, we did not have any quantitative means to pair them between the two brain-mapping/imaging systems (i.e., EEG and fMRI). Also, the EEG electrodes in our study were systematically moved ∼1–1.5 cm toward the back of the head; that would generate certain shifts in the 2D and/or 3D EEG brain networks. This physical shift added uncertainty for good matches between our SVD-derived EEG brain network and conventional fMRI-created brain network. Thus, concurrent EEG-fMRI recording along with tPBM would be an ideal experimental design, with high technical challenges, that enables to confirm or better understand the relationships between tPBM and responsive brain networks in the human brain. Second, in this study, the source localization was performed based on 64 electrodes of EEG, which might not be adequate to provide high spatial resolution for accurate localization of cortical sources. For example, although we provided evidence of temporal independency of the first three SVD components that had close-to-zero correlations between their time dynamics, we were unable to spatially separate their cortical active regions with eLORETA, mainly caused by the low resolution in 3D source localization. Nevertheless, this inseparable sources among the three SVD components do not diminish the feasibility and correctness of SVD-derived components. This is because the mathematical rigor of the SVD algorithm warrants to accurately decompose multi-electrode EEG into independent components (i.e., 2D topographies) in the EEG sensor space. One solution is to take high-density EEG measures with 128 or 256 electrodes in the future, which can improve the spatial resolution of 3D source localization.

## Conclusion

We implemented gSVD as a novel methodology to analyze human EEG signal, followed by eLORETA source localization, two of which together facilitated the novel identification of 11 independent and orthogonal EEG brain networks in both EEG sensor and source spaces. Following a sham-controlled experimental protocol, we found that right-forehead, 1,064-nm tPBM could neuromodulate the alpha and gamma powers on several of the gSVD-derived EEG brain networks. Moreover, many similarities were observed/found between the EEG cortical networks and fMRI-recognized networks, demonstrating that prefrontal tPBM can neuromodulate the well-defined (i.e., MRI-derived) default-mode network, frontal-parietal network, and executive control network. These results also clearly reveal mechanistic associations or causal effects of tPBM and modulated brain networks versus improved cognition outcomes.

## Data Availability Statement

The raw data supporting the conclusions of this article will be made available by the authors upon request, without undue reservation.

## Ethics Statement

The studies involving human participants were reviewed and approved by the Institutional Review Board of the University of Texas at Arlington. The patients/participants provided their written informed consent to participate in this study.

## Author Contributions

XW analyzed the data, interpreted the results, and prepared the manuscript. HW recruited human participants, collected experimental data, and managed/organized the data. AW assisted the data collection, discussed the results, and reviewed the manuscript. HL initiated and supervised the study, discussed and interpreted the results, as well as reviewed and revised the manuscript.

## Conflict of Interest

The authors declare that the research was conducted in the absence of any commercial or financial relationships that could be construed as a potential conflict of interest. The handling editor FG-L declared a past co-authorship with the authors.

## Publisher’s Note

All claims expressed in this article are solely those of the authors and do not necessarily represent those of their affiliated organizations, or those of the publisher, the editors and the reviewers. Any product that may be evaluated in this article, or claim that may be made by its manufacturer, is not guaranteed or endorsed by the publisher.

## Footnotes

1
http://www.uzh.ch/keyinst/loreta.htm

## References

[B1] Akiki T. J. Averill C. L. Wrocklage K. M. Scott J. C. Averill L. A. Schweinsburg B. (2018). Default mode network abnormalities in posttraumatic stress disorder: a novel network-restricted topology approach. *Neuroimage* 176 489–498. 10.1016/j.neuroimage.2018.05.005 29730491 PMC5976548

[B2] Angelakis E. Stathopoulou S. Frymiare J. L. Green D. L. Lubar J. F. Kounios J. (2007). EEG neurofeedback: a brief overview and an example of peak alpha frequency training for cognitive enhancement in the elderly. *Clin. Neuropsychol.* 21 110–129. 10.1080/13854040600744839 17366280

[B3] Aoki Y. Ishii R. Pascual-Marqui R. D. Canuet L. Ikeda S. Hata M. (2015). Detection of EEG-resting state independent networks by eLORETA-ICA method. *Front. Hum. Neurosci.* 9:31. 10.3389/fnhum.2015.00031 25713521 PMC4322703

[B4] Asadi N. Wang Y. Olson I. Obradovic Z. (2020). A heuristic information cluster search approach for precise functional brain mapping. *Hum. Brain Mapp.* 41 2263–2280. 10.1002/hbm.24944 32034846 PMC7267912

[B5] Bai M. Huang Y. Zhang G. Zheng W. Lin Q. Hu Z. (2019). Fast backward singular value decomposition (SVD) algorithm for magnetocardiographic signal reconstruction from pulsed atomic magnetometer data. *Opt. Express* 27 29534–29546. 10.1364/OE.27.029534 31684213

[B6] Barrett D. W. Gonzalez-Lima F. (2013). Transcranial infrared laser stimulation produces beneficial cognitive and emotional effects in humans. *Neuroscience* 230 13–23. 10.1016/j.neuroscience.2012.11.016 23200785

[B7] Bender P. Zakutna D. Disch S. Marcano L. Alba Venero D. Honecker D. (2019). Using the singular value decomposition to extract 2D correlation functions from scattering patterns. *Acta Crystallogr. A Found. Adv.* 75 766–771. 10.1107/S205327331900891X 31475920

[B8] Berman M. H. Halper J. P. Nichols T. W. Jarrett H. Lundy A. Huang J. H. (2017). Photobiomodulation with near infrared light helmet in a pilot, placebo controlled clinical trial in dementia patients testing memory and cognition. *J. Neurol. Neurosci.* 8:176. 10.21767/2171-6625.1000176 28593105 PMC5459322

[B9] Binder J. R. Desai R. H. (2011). The neurobiology of semantic memory. *Trends Cogn. Sci.* 15 527–536. 10.1016/j.tics.2011.10.001 22001867 PMC3350748

[B10] Blanco N. J. Maddox W. T. Gonzalez-Lima F. (2015). Improving executive function using transcranial infrared laser stimulation. *J. Neuropsychol.* 11 14–25. 10.1111/jnp.12074 26017772 PMC4662930

[B11] Blanco N. J. Saucedo C. L. Gonzalez-Lima F. (2017). Transcranial infrared laser stimulation improves rule-based, but not information-integration, category learning in humans. *Neurobiol. Learn. Mem.* 139 69–75. 10.1016/j.nlm.2016.12.016 28039085

[B12] Buckner R. L. Andrews-Hanna J. R. Schacter D. L. (2008). The brain’s default network: anatomy, function, and relevance to disease. *Ann. N. Y. Acad. Sci.* 1124 1–38. 10.1196/annals.1440.011 18400922

[B13] Calhoun V. D. Liu J. Adali T. (2009). A review of group ICA for fMRI data and ICA for joint inference of imaging, genetic, and ERP data. *Neuroimage* 45(Suppl. 1) S163–S172. 10.1016/j.neuroimage.2008.10.057 19059344 PMC2651152

[B14] Candès E. J. Li X. Ma Y. Wright J. (2011). Robust principal component analysis? *J. ACM* 58 1–37.

[B15] Chan A. S. Lee T. L. Hamblin M. R. Cheung M. C. (2021). Photobiomodulation enhances memory processing in older adults with mild cognitive impairment: a functional near-infrared spectroscopy study. *J. Alzheimers Dis.* 83 1471–1480. 10.3233/JAD-201600 33998541

[B16] Chan R. C. Shum D. Toulopoulou T. Chen E. Y. (2008). Assessment of executive functions: review of instruments and identification of critical issues. *Arch. Clin. Neuropsychol.* 23 201–216. 10.1016/j.acn.2007.08.010 18096360

[B17] Chaudhari A. Wang X. Wu A. Dmochowski J. P. Liu H. (2021). “Transcranial photobiomodulation with light emitting diodes increases vigilance performance and EEG alpha power of the human brain,” in *Proceedings of the Novel Techniques in Microscopy:* JW1A.20, (Washington, DC: Optical Society of America).

[B18] Chen X. C. Litvinov Y. A. Wang M. Wang Q. Zhang Y. H. (2019). Denoising scheme based on singular-value decomposition for one-dimensional spectra and its application in precision storage-ring mass spectrometry. *Phys. Rev. E* 99:063320. 10.1103/PhysRevE.99.063320 31330675

[B19] Chowdhury S. R. Dutta J. (2019). Higher-order singular value decomposition-based lung parcellation for breathing motion management. *J. Med. Imaging (Bellingham)* 6:024004. 10.1117/1.JMI.6.2.024004 31065568 PMC6499405

[B20] Clements G. M. Bowie D. C. Gyurkovics M. Low K. A. Fabiani M. Gratton G. (2021). Spontaneous alpha and theta oscillations are related to complementary aspects of cognitive control in younger and older adults. *Front. Hum. Neurosci.* 15:621620. 10.3389/fnhum.2021.621620 33841114 PMC8025241

[B21] Cohen M. (2021). *Data Analysis Lecturelets.* Available online at: http://mikexcohen.com/lectures.html (accessed January 2021).

[B22] Cohen M. X. (2014). *Analyzing Neural Time Series Data: Theory and Practice.* Cambridge, MA: The MIT Press.

[B23] Collins A. Koechlin E. (2012). Reasoning, learning, and creativity: frontal lobe function and human decision-making. *PLoS Biol.* 10:e1001293. 10.1371/journal.pbio.1001293 22479152 PMC3313946

[B24] Custo A. Van De Ville D. Wells W. M. Tomescu M. I. Brunet D. Michel C. M. (2017). Electroencephalographic resting-state networks: source localization of microstates. *Brain Connect.* 7 671–682. 10.1089/brain.2016.0476 28938855 PMC5736178

[B25] Dastjerdi M. Foster B. L. Nasrullah S. Rauschecker A. M. Dougherty R. F. Townsend J. D. (2011). Differential electrophysiological response during rest, self-referential, and non–self-referential tasks in human posteromedial cortex. *Proc. Natl. Acad. Sci. U.S.A.* 108 3023–3028. 10.1073/pnas.1017098108 21282630 PMC3041085

[B26] Delorme A. Makeig S. (2004). EEGLAB: an open source toolbox for analysis of single-trial EEG dynamics including independent component analysis. *J. Neurosci. Methods* 134 9–21. 10.1016/j.jneumeth.2003.10.009 15102499

[B27] Diamond A. (2013). Executive functions. *Annu. Rev. Psychol.* 64 135–168. 10.1146/annurev-psych-113011-143750 23020641 PMC4084861

[B28] Dougal G. Ennaceur A. Chazot P. L. (2021). Effect of transcranial near-infrared light 1068 nm upon memory performance in aging healthy individuals: a pilot study. *Photobiomodul. Photomed. Laser Surg.* 39 654–660. 10.1089/photob.2020.4956 34662523

[B29] Eells J. T. Wong-Riley M. T. VerHoeve J. Henry M. Buchman E. V. Kane M. P. (2004). Mitochondrial signal transduction in accelerated wound and retinal healing by near-infrared light therapy. *Mitochondrion* 4 559–567. 10.1016/j.mito.2004.07.033 16120414

[B30] Euston D. R. Gruber A. J. McNaughton B. L. (2012). The role of medial prefrontal cortex in memory and decision making. *Neuron* 76 1057–1070. 10.1016/j.neuron.2012.12.002 23259943 PMC3562704

[B31] Fitzgerald P. J. Watson B. O. (2018). Gamma oscillations as a biomarker for major depression: an emerging topic. *Transl. Psychiatry* 8:177. 10.1038/s41398-018-0239-y 30181587 PMC6123432

[B32] Ghaderi A. H. Jahan A. Akrami F. Moghadam Salimi M. (2021). Transcranial photobiomodulation changes topology, synchronizability, and complexity of resting state brain networks. *J. Neural Eng.* 18 046048. 10.1088/1741-2552/abf97c 33873167

[B33] Ginebreda A. Sabater-Liesa L. Barcelo D. (2019). Quantification of ecological complexity and resilience from multivariate biological metrics datasets using singular value decomposition entropy. *MethodsX* 6 1668–1676. 10.1016/j.mex.2019.07.020 31384567 PMC6664095

[B34] Gonzalez-Lima F. Auchter A. (2015). Protection against neurodegeneration with low-dose methylene blue and near-infrared light. *Front. Cell. Neurosci.* 9:179. 10.3389/fncel.2015.00179 26029050 PMC4428125

[B35] Gonzalez-Lima F. Barrett D. W. (2014). Augmentation of cognitive brain functions with transcranial lasers. *Front. Syst. Neurosci.* 8:36. 10.3389/fnsys.2014.00036 24672439 PMC3953713

[B36] Grill-Spector K. Kourtzi Z. Kanwisher N. (2001). The lateral occipital complex and its role in object recognition. *Vis. Res.* 41 1409–1422. 10.1016/s0042-6989(01)00073-6 11322983

[B37] Guillemot V. Beaton D. Gloaguen A. Löfstedt T. Levine B. Raymond N. (2019). A constrained singular value decomposition method that integrates sparsity and orthogonality. *PLoS One* 14:e0211463. 10.1371/journal.pone.0211463 30865639 PMC6415851

[B38] Haddad A. E. Najafizadeh L. (2015). “Global EEG segmentation using singular value decomposition,” in *Proceedings of the 2015, 37th Annual International Conference of the IEEE Engineering in Medicine and Biology Society (EMBC)*, (Milan: IEEE), 558–561. 10.1109/EMBC.2015.7318423 26736323

[B39] Hamblin M. R. (2016). Shining light on the head: photobiomodulation for brain disorders. *BBA Clin.* 6 113–124. 10.1016/j.bbacli.2016.09.002 27752476 PMC5066074

[B40] Hamblin M. R. Huang Y. Y. (eds) (2019). *Photobiomodulation in the Brain.* San Diago, CA: Acamemic press.

[B41] Harner R. N. (1990). Singular value decomposition—A general linear model for analysis of multivariate structure in the electroencephalogram. *Brain Topogr.* 3 43–47. 10.1007/BF01128860 2094312

[B42] Henderson T. A. Morries L. D. (2015). Near-infrared photonic energy penetration: can infrared phototherapy effectively reach the human brain? *Neuropsychiatr. Dis. Treat.* 11 2191–2208. 10.2147/NDT.S78182 26346298 PMC4552256

[B43] Huotari N. Raitamaa L. Helakari H. Kananen J. Raatikainen V. Rasila A. (2019). Sampling rate effects on resting state fMRI metrics. *Front. Neurosci.* 13:279. 10.3389/fnins.2019.00279 31001071 PMC6454039

[B44] Hwang J. Castelli D. M. Gonzalez-Lima F. (2016). Cognitive enhancement by transcranial laser stimulation and acute aerobic exercise. *Lasers Med. Sci.* 31 1151–1160. 10.1007/s10103-016-1962-3 27220529

[B45] Ikeda S. Ishii R. Pascual-Marqui R. D. Canuet L. Yoshimura M. Nishida K. (2019). Automated source estimation of scalp EEG epileptic activity using eLORETA kurtosis analysis. *Neuropsychobiology* 77 101–109. 10.1159/000495522 30625490

[B46] Imperatori C. Fabbricatore M. Innamorati M. Farina B. Quintiliani M. I. Lamis D. A. (2015). Modification of EEG functional connectivity and EEG power spectra in overweight and obese patients with food addiction: an eLORETA study. *Brain Imaging Behav.* 9 703–716. 10.1007/s11682-014-9324-x 25332109

[B47] Iriarte J. Urrestarazu E. Valencia M. Alegre M. Malanda A. Viteri C. (2003). Independent component analysis as a tool to eliminate artifacts in EEG: a quantitative study. *J. Clin. Neurophysiol.* 20 249–257. 10.1097/00004691-200307000-00004 14530738

[B48] Jagdeo J. R. Adams L. E. Brody N. I. Siegel D. M. (2012). Transcranial red and near infrared light transmission in a cadaveric model. *PLoS One* 7:e47460. 10.1371/journal.pone.0047460 23077622 PMC3471828

[B49] Jahan A. Nazari M. A. Mahmoudi J. Salehpour F. Salimi M. M. (2019). Transcranial near-infrared photobiomodulation could modulate brain electrophysiological features and attentional performance in healthy young adults. *Lasers Med. Sci.* 34 1193–1200. 10.1007/s10103-018-02710-3 31011865

[B50] Janicak P. G. (2002). Molecular neuropharmacology: a foundation for clinical neuroscience. *Am. J. Psychiatry* 159 1251–1251. 10.1176/appi.ajp.159.7.1251

[B51] Jann K. Kottlow M. Dierks T. Boesch C. Koenig T. (2010). Topographic electrophysiological signatures of FMRI resting state networks. *PLoS One* 5:e12945. 10.1371/journal.pone.0012945 20877577 PMC2943931

[B52] Jatoi M. A. Kamel N. Malik A. S. Faye I. (2014). EEG based brain source localization comparison of sLORETA and eLORETA. *Australas. Phys. Eng. Sci. Med.* 37 713–721. 10.1007/s13246-014-0308-3 25359588

[B53] Jonmohamadi Y. Forsyth A. McMillan R. Muthukumaraswamy S. D. (2019). Constrained temporal parallel decomposition for EEG-fMRI fusion. *J. Neural Eng.* 16:016017. 10.1088/1741-2552/aaefda 30523889

[B54] Kawasaki M. Kitajo K. Yamaguchi Y. (2010). Dynamic links between theta executive functions and alpha storage buffers in auditory and visual working memory. *Eur. J. Neurosci.* 31 1683–1689. 10.1111/j.1460-9568.2010.07217.x 20525081 PMC2878597

[B55] Koenigs M. Barbey A. K. Postle B. R. Grafman J. (2009). Superior parietal cortex is critical for the manipulation of information in working memory. *J. Neurosci.* 29 14980–14986. 10.1523/JNEUROSCI.3706-09.2009 19940193 PMC2799248

[B56] Kozlovskiy S. A. Nikonova E. Y. Pyasik M. M. Velichkovsky B. M. (2012). The cingulate cortex and human memory processes. *Psychol. Russia* 5 231. 10.11621/pir.2012.0014

[B57] Krol L. R. (2021). *Permutation Test*, ed. Mathworks Available online at: https://www.mathworks.com/matlabcentral/fileexchange/63276-permutation-test (accessed March 2021).

[B58] Lagerlund T. D. Sharbrough F. W. Busacker N. E. (1997). Spatial filtering of multichannel electroencephalographic recordings through principal component analysis by singular value decomposition. *J. Clin. Neurophysiol.* 14 73–82. 10.1097/00004691-199701000-00007 9013362

[B59] Lampl Y. Zivin J. A. Fisher M. Lew R. Welin L. Dahlof B. (2007). Infrared laser therapy for ischemic stroke: a new treatment strategy: results of the NeuroThera Effectiveness and Safety Trial-1 (NEST-1). *Stroke* 38 1843–1849. 10.1161/STROKEAHA.106.478230 17463313

[B60] Leech R. Sharp D. J. (2014). The role of the posterior cingulate cortex in cognition and disease. *Brain* 137(Pt 1) 12–32. 10.1093/brain/awt162 23869106 PMC3891440

[B61] Lefebvre E. D’Angiulli A. (2019). Imagery-mediated verbal learning depends on vividness–familiarity interactions: the possible role of dualistic resting state network activity interference. *Brain Sci.* 9:143. 10.3390/brainsci9060143 31216699 PMC6627679

[B62] Li S. Eloyan A. Joel S. Mostofsky S. Pekar J. Bassett S. S. (2012). Analysis of group ICA-based connectivity measures from fMRI: application to Alzheimer’s disease. *PLoS One* 7:e49340. 10.1371/journal.pone.0049340 23226208 PMC3511486

[B63] Lundstrom B. N. Petersson K. M. Andersson J. Johansson M. Fransson P. Ingvar M. (2003). Isolating the retrieval of imagined pictures during episodic memory: activation of the left precuneus and left prefrontal cortex. *Neuroimage* 20 1934–1943. 10.1016/j.neuroimage.2003.07.017 14683699

[B64] Manly B. F. J. (2007). *Randomization, Bootstrap and Monte Carlo Methods in Biology*, 3rd Edn. Boca Raton, FL: Chapman and Hall/CRC.

[B65] Mantini D. Perrucci M. G. Del Gratta C. Romani G. L. Corbetta M. (2007). Electrophysiological signatures of resting state networks in the human brain. *Proc. Natl. Acad. Sci. U.S.A.* 104 13170–13175. 10.1073/pnas.0700668104 17670949 PMC1941820

[B66] Marek S. Dosenbach N. U. F. (2018). The frontoparietal network: function, electrophysiology, and importance of individual precision mapping. *Dialogues Clin. Neurosci.* 20 133–140. 10.31887/DCNS.2018.20.2/smarek 30250390 PMC6136121

[B67] Mohan A. Roberto A. J. Mohan A. Lorenzo A. Jones K. Carney M. J. (2016). The significance of the default mode network (DMN) in neurological and neuropsychiatric disorders: a review. *Yale J. Biol. Med.* 89 49–57. 27505016 PMC4797836

[B68] Murphy C. Jefferies E. Rueschemeyer S. A. Sormaz M. Wang H. T. Margulies D. S. (2018). Distant from input: evidence of regions within the default mode network supporting perceptually-decoupled and conceptually-guided cognition. *Neuroimage* 171 393–401. 10.1016/j.neuroimage.2018.01.017 29339310 PMC5883322

[B69] Nielsen J. D. Madsen K. H. Wang Z. Liu Z. Friston K. J. Zhou Y. (2017). Working memory modulation of frontoparietal network connectivity in first-episode schizophrenia. *Cereb. Cortex* 27 3832–3841. 10.1093/cercor/bhx050 28334138

[B70] Nizamutdinov D. Qi X. Berman M. H. Dougal G. Dayawansa S. Wu E. (2021). Transcranial near infrared light stimulations improve cognition in patients with dementia. *Aging Dis.* 12 954–963. 10.14336/AD.2021.0229 34221541 PMC8219492

[B71] Numssen O. Bzdok D. Hartwigsen G. (2021). Functional specialization within the inferior parietal lobes across cognitive domains. *ELife* 10:e63591. 10.7554/eLife.63591 33650486 PMC7946436

[B72] Osipova D. Hermes D. Jensen O. (2008). Gamma power is phase-locked to posterior alpha activity. *PLoS One* 3:e3990. 10.1371/journal.pone.0003990 19098986 PMC2602598

[B73] Pascual-Marqui R. D. Lehmann D. Koukkou M. Kochi K. Anderer P. Saletu B. (2011). Assessing interactions in the brain with exact low-resolution electromagnetic tomography. *Philos. Trans. A Math. Phys. Eng. Sci.* 369 3768–3784. 10.1098/rsta.2011.0081 21893527

[B74] Piano C. Imperatori C. Losurdo A. Bentivoglio A. R. Cortelli P. Della Marca G. (2017). Sleep-related modifications of EEG connectivity in the sensory-motor networks in Huntington Disease: an eLORETA study and review of the literature. *Clin. Neurophysiol.* 128 1354–1363. 10.1016/j.clinph.2016.11.019 28043770

[B75] Pruitt T. Wang X. Wu A. Kallioniemi E. Husain M. M. Liu H. (2020). Transcranial photobiomodulation (tPBM) with 1,064-nm laser to improve cerebral metabolism of the human brain in vivo. *Lasers Surg. Med.* 52 807–813. 10.1002/lsm.23232 32173886 PMC7492377

[B76] Raichle M. E. Snyder A. Z. (2007). A default mode of brain function: a brief history of an evolving idea. *Neuroimage* 37 1083–1090; discussion1097–1099. 10.1016/j.neuroimage.2007.02.041 17719799

[B77] Ramkumar P. Parkkonen L. Hari R. Hyvarinen A. (2012). Characterization of neuromagnetic brain rhythms over time scales of minutes using spatial independent component analysis. *Hum. Brain Mapp.* 33 1648–1662. 10.1002/hbm.21303 21915941 PMC6869857

[B78] Renier L. A. Anurova I. De Volder A. G. Carlson S. VanMeter J. Rauschecker J. P. (2010). Preserved functional specialization for spatial processing in the middle occipital gyrus of the early blind. *Neuron* 68 138–148. 10.1016/j.neuron.2010.09.021 20920797 PMC2951740

[B79] Rojas J. C. Gonzalez-Lima F. (2011). Low-level light therapy of the eye and brain. *Eye Brain* 3 49–67. 10.2147/EB.S21391 28539775 PMC5436183

[B80] Rojas J. C. Gonzalez-Lima F. (2013). Neurological and psychological applications of transcranial lasers and LEDs. *Biochem. Pharmacol.* 86 447–457. 10.1016/j.bcp.2013.06.012 23806754

[B81] Saucedo C. L. Courtois E. C. Wade Z. S. Kelley M. N. Kheradbin N. Barrett D. W. (2021). Transcranial laser stimulation: mitochondrial and cerebrovascular effects in younger and older healthy adults. *Brain Stimul.* 14 440–449. 10.1016/j.brs.2021.02.011 33636401

[B82] Shahid A. Kamel N. Malik A. S. (2014). “Singular values as a detector of epileptic seizures in EEG signals,” in *Proceedings of the 2014 5th International Conference on Intelligent and Advanced Systems (ICIAS)*, (Kuala Lumpur: IEEE), 1–5.

[B83] Shen H. H. (2015). Core concept: resting-state connectivity. *Proc. Natl. Acad. Sci. U.S.A.* 112 14115–14116. 10.1073/pnas.1518785112 26578753 PMC4655520

[B84] Snyder D. B. Schmit B. D. Hyngstrom A. S. Beardsley S. A. (2021). Electroencephalography resting-state networks in people with Stroke. *Brain Behav.* 11:e02097. 10.1002/brb3.2097 33759382 PMC8119848

[B85] Sormaz M. Murphy C. Wang H. T. Hymers M. Karapanagiotidis T. Poerio G. (2018). “Default mode network can support the level of detail in experience during active task states”: correction. *Proc. Natl. Acad. Sci. U.S.A.* 115 9318–9323. 10.1073/pnas.1817966115 30150393 PMC6140531

[B86] Spera V. Sitnikova T. Ward M. J. Farzam P. Hughes J. Gazecki S. (2021). Pilot study on dose-dependent effects of transcranial photobiomodulation on brain electrical oscillations: a potential therapeutic target in Alzheimer’s disease. *J. Alzheimers Dis.* 83 1481–1498. 10.3233/JAD-210058 34092636

[B87] Stevens F. L. Hurley R. A. Taber K. H. (2011). Anterior cingulate cortex: unique role in cognition and emotion. *J. Neuropsychiatry Clin. Neurosci.* 23 121–125. 10.1176/appi.neuropsych.23.2.121 21677237

[B88] Tagliazucchi E. von Wegner F. Morzelewski A. Brodbeck V. Laufs H. (2012). Dynamic BOLD functional connectivity in humans and its electrophysiological correlates. *Front. Hum. Neurosci.* 6:339. 10.3389/fnhum.2012.00339 23293596 PMC3531919

[B89] Tanaka-Koshiyama K. Koshiyama D. Miyakoshi M. Joshi Y. B. Molina J. L. Sprock J. (2020). Abnormal spontaneous gamma power is associated with verbal learning and memory dysfunction in schizophrenia. *Front. Psychiatry* 11:832. 10.3389/fpsyt.2020.00832 33110410 PMC7488980

[B90] Tedford C. E. DeLapp S. Jacques S. Anders J. (2015). Re: “Quantitative analysis of transcranial and intraparenchymal light penetration in human cadaver brain tissue” Lasers in Surgery and Medicine, 2015;47(4):312-322. *Lasers Surg. Med.* 47:466. 10.1002/lsm.22377 25772014

[B91] Teng C. Zhou J. Ma H. Tan Y. Wu X. Guan C. (2018). Abnormal resting state activity of left middle occipital gyrus and its functional connectivity in female patients with major depressive disorder. *BMC Psychiatry* 18:370. 30477561 10.1186/s12888-018-1955-9 PMC6258168

[B92] Tops M. Boksem M. A. (2011). A potential role of the inferior frontal gyrus and anterior insula in cognitive control, brain rhythms, and event-related potentials. *Front. Psychol.* 2:330. 10.3389/fpsyg.2011.00330 22084637 PMC3212750

[B93] Turnip A. (2014). “Automatic artifacts removal of EEG signals using robust principal component analysis,” in *Proceedings of the 2014 2nd International Conference on Technology, Informatics, Management, Engineering & Environment*, (Bandung: IEEE), 331–334.

[B94] Urquhart E. L. Wanniarachchi H. Wang X. Gonzalez-Lima F. Alexandrakis G. Liu H. (2020). Transcranial photobiomodulation-induced changes in human brain functional connectivity and network metrics mapped by whole-head functional near-infrared spectroscopy in vivo. *Biomed. Opt. Express* 11 5783–5799. 10.1364/BOE.402047 33149986 PMC7587286

[B95] Vargas E. Barrett D. W. Saucedo C. L. Huang L. D. Abraham J. A. Tanaka H. (2017). Beneficial neurocognitive effects of transcranial laser in older adults. *Lasers Med. Sci.* 32 1153–1162. 10.1007/s10103-017-2221-y 28466195 PMC6802936

[B96] Veer I. M. Beckmann C. F. van Tol MJ Ferrarini L. Milles J. Veltman D. J. (2010). Whole brain resting-state analysis reveals decreased functional connectivity in major depression. *Front. Syst. Neurosci.* 4:41. 10.3389/fnsys.2010.00041 20941370 PMC2950744

[B97] Voytek B. Canolty R. T. Shestyuk A. Crone N. E. Parvizi J. Knight R. T. (2010). Shifts in gamma phase-amplitude coupling frequency from theta to alpha over posterior cortex during visual tasks. *Front. Hum. Neurosci.* 4:191. 10.3389/fnhum.2010.00191 21060716 PMC2972699

[B98] Wall M. E. Rechtsteiner A. Rocha L. M. (2003). “Singular value decomposition and principal component analysis,” in *A practical Approach to Microarray Data Analysis*, eds Berrar D. P. Dubitzky W. Granzow M. (Boston, MA: Springer), 91–109. 10.1007/0-306-47815-3_5

[B99] Wallentin M. Roepstorff A. Glover R. Burgess N. (2006). Parallel memory systems for talking about location and age in precuneus, caudate and Broca’s region. *Neuroimage* 32 1850–1864. 10.1016/j.neuroimage.2006.05.002 16828565

[B100] Wang X. Dmochowski J. P. Zeng L. Kallioniemi E. Husain M. Gonzalez-Lima F. (2019). Transcranial photobiomodulation with 1064-nm laser modulates brain electroencephalogram rhythms. *Neurophotonics* 6:025013. 10.1117/1.NPh.6.2.025013 31259198 PMC6563945

[B101] Wang X. Dmochowski J. Husain M. Gonzalez-Lima F. Liu H. (2017). Proceedings# 18. Transcranial infrared brain stimulation modulates EEG alpha power. *Brain Stimul.* 10 e67–e69.

[B102] Wang X. Reddy D. D. Nalawade S. S. Pal S. Gonzalez-Lima F. Liu H. (2018). Impact of heat on metabolic and hemodynamic changes in transcranial infrared laser stimulation measured by broadband near-infrared spectroscopy. *Neurophotonics* 5:011004. 10.1117/1.NPh.5.1.011004 28948191 PMC5603720

[B103] Wang X. Tian F. Soni S. S. Gonzalez-Lima F. Liu H. (2016). Interplay between up-regulation of cytochrome-c-oxidase and hemoglobin oxygenation induced by near-infrared laser. *Sci. Rep.* 6:30540. 10.1038/srep30540 27484673 PMC4971496

[B104] Wang X. Wanniarachchi H. Wu A. Gonzalez-Lima F. Liu H. (2021). Transcranial photobiomodulation and thermal stimulation induce distinct topographies of EEG alpha and beta power changes in healthy humans. *Sci. Rep.* 11:18917. 10.1038/s41598-021-97987-w 34556692 PMC8460746

[B105] Wong-Riley M. T. Liang H. L. Eells J. T. Chance B. Henry M. M. Buchmann E. (2005). Photobiomodulation directly benefits primary neurons functionally inactivated by toxins: role of cytochrome c oxidase. *J. Biol. Chem.* 280 4761–4771. 10.1074/jbc.M409650200 15557336

[B106] Wu Q. Wang X. Liu H. Zeng L. (2019). Learning hemodynamic effect of transcranial infrared laser stimulation using longitudinal data analysis. *IEEE J. Biomed. Health Inform.* 24 1772–1779. 10.1109/JBHI.2019.2951772 31714245 PMC7316150

[B107] Yao L. Qian Z. Liu Y. Fang Z. Li W. Xing L. (2020). Effects of stimulating frequency of NIR LEDs light irradiation on forehead as quantified by EEG measurements. *J. Innov. Opt. Health Sci.* 14:2050025. 10.1142/s179354582050025x

[B108] Yuen N. H. Osachoff N. Chen J. J. (2019). Intrinsic frequencies of the resting-state fMRI signal: the frequency dependence of functional connectivity and the effect of mode mixing. *Front. Neurosci.* 13:900. 10.3389/fnins.2019.00900 31551676 PMC6738198

[B109] Zhou R. Wang J. Qi W. Liu F. Y. Yi M. Guo H. (2018). Elevated resting state gamma oscillatory activities in electroencephalogram of patients with post-herpetic neuralgia. *Front. Neurosci.* 12:750. 10.3389/fnins.2018.00750 30405337 PMC6205978

[B110] Zivin J. A. Albers G. W. Bornstein N. Chippendale T. Dahlof B. Devlin T. (2009). Effectiveness and safety of transcranial laser therapy for acute ischemic stroke. *Stroke* 40 1359–1364. 10.1161/STROKEAHA.109.547547 19233936

[B111] Zomorrodi R. Loheswaran G. Pushparaj A. Lim L. (2019). Pulsed near infrared transcranial and intranasal photobiomodulation significantly modulates neural oscillations: a pilot exploratory study. *Sci. Rep.* 9:6309. 10.1038/s41598-019-42693-x 31004126 PMC6474892

