# Supplementary material for: Combination of Group Singular Value Decomposition and eLORETA Identifies Human EEG Networks and Responses to Transcranial Photobiomodulation
Source: Front Hum Neurosci. 2022 May 10;16:853909. doi: 10.3389/fnhum.2022.853909 (PMC9127055; doi:10.3389/fnhum.2022.853909)
Supplement: Supplementary file 1 [file Data_Sheet_1.docx]

Supplementary materials


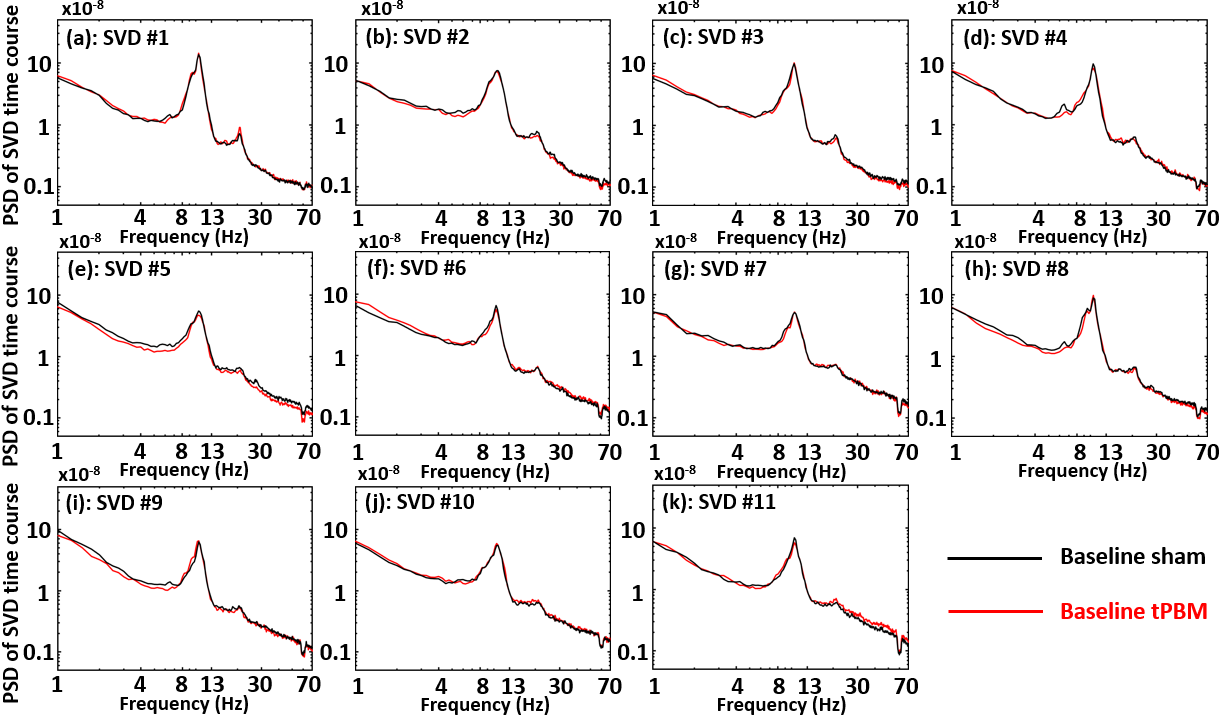


**Figure 1** (a)-(k) PSD curves calculated from the temporal series of SVD #1-11 during 2-min baseline periods before starting sham (black) or tPBM (red) stimulations from n=44 subjects.


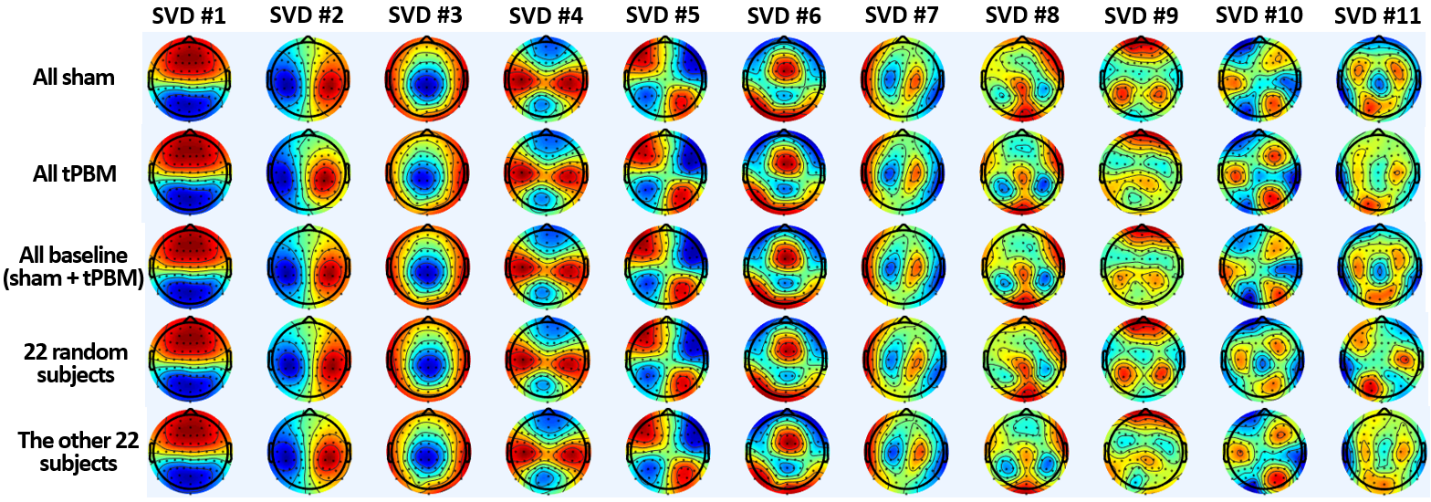


**Figure 2** Highly repeatable topographical patterns obtained by gSVD using “44 data sets from sham measurements through 11-min recordings” (1^st^ row), “44 data sets from tPBM-active measurements through 11-min recordings” (2^nd^ row), “88 data sets of 2-min baseline data” (3^rd^ row), “random selection of 22 participants through 11-min period” (4^th^ row), and “random selection of the other 22 participants through 11-min period”.


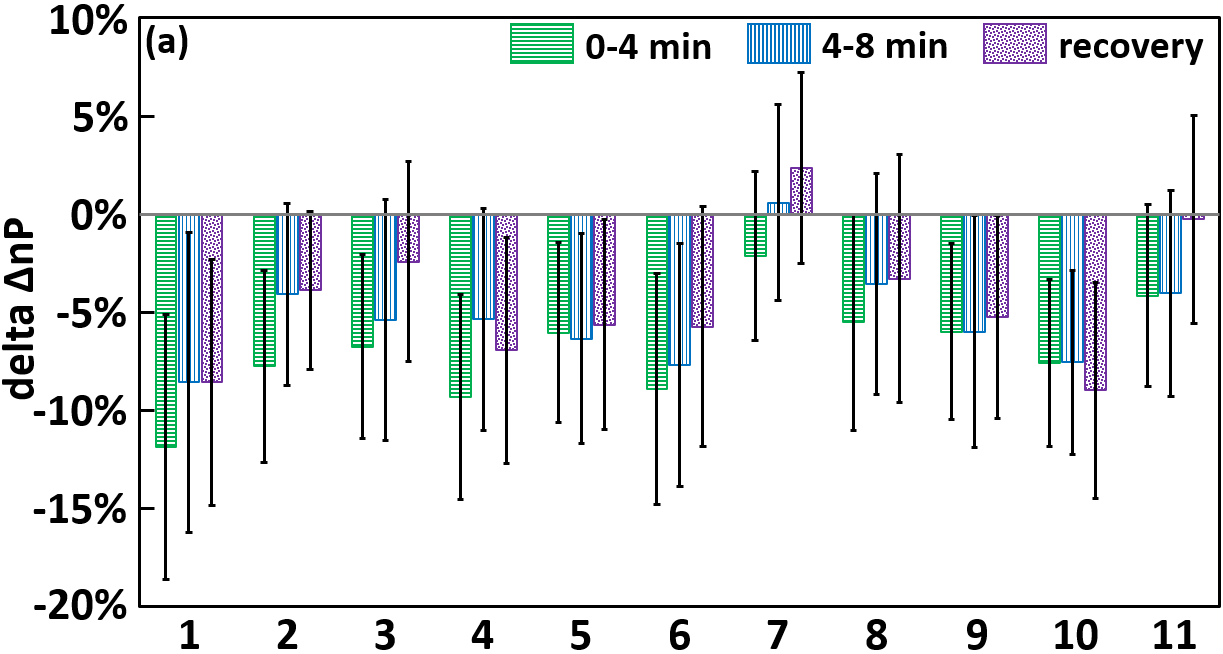

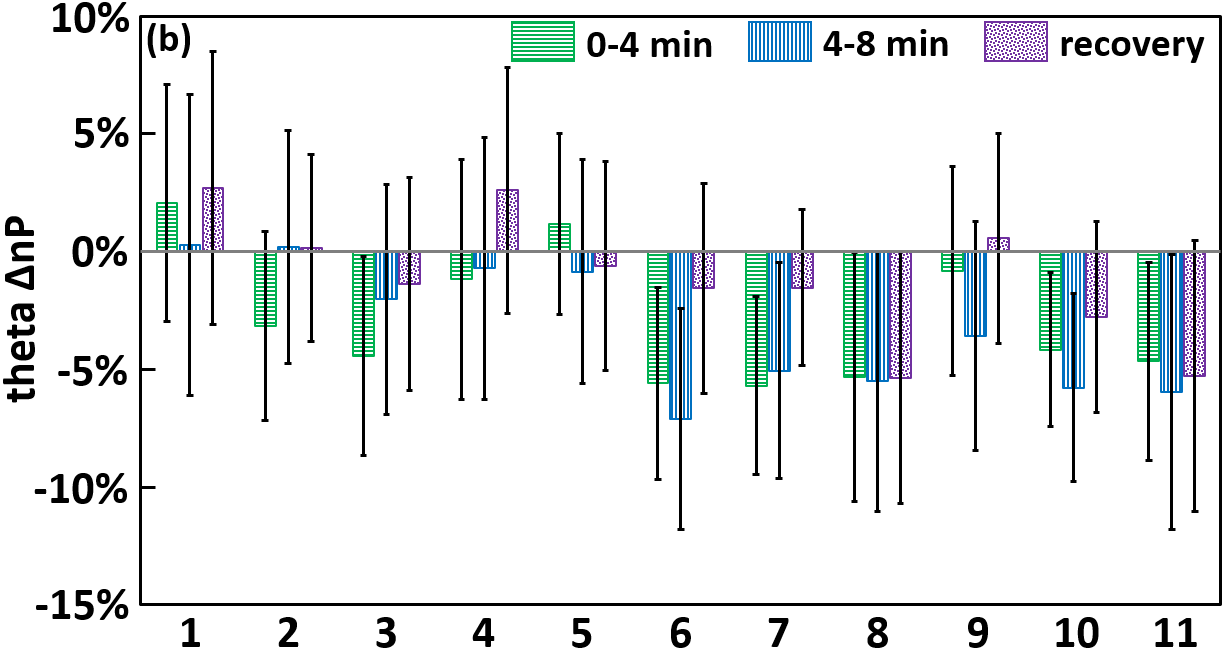

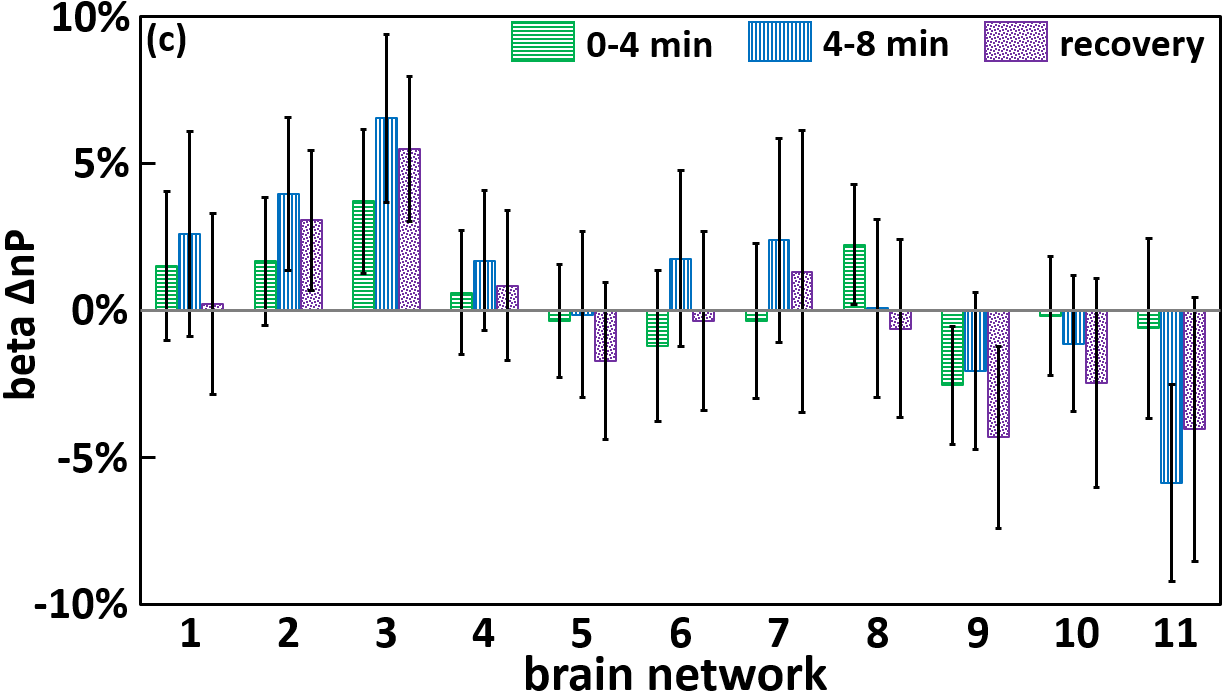


**Figure 3** Group-level (n=44) ΔnP for each brain network in (a) delta, (b) theta, and (c) beta bands, during the 0-4 min (green horizontal strip bars), 4-8 min (blue vertical strip bars) of tPBM/sham, and recovery (purple dotted bars) periods. No significant differences between each bar versus zero was observed when one-sample non-parametric tests were taken between ΔnP versus zero at the significance level of p < 0.05. This is equivalent to a two-sample pair-wise non-parametric test between nP values of tPBM versus sham.


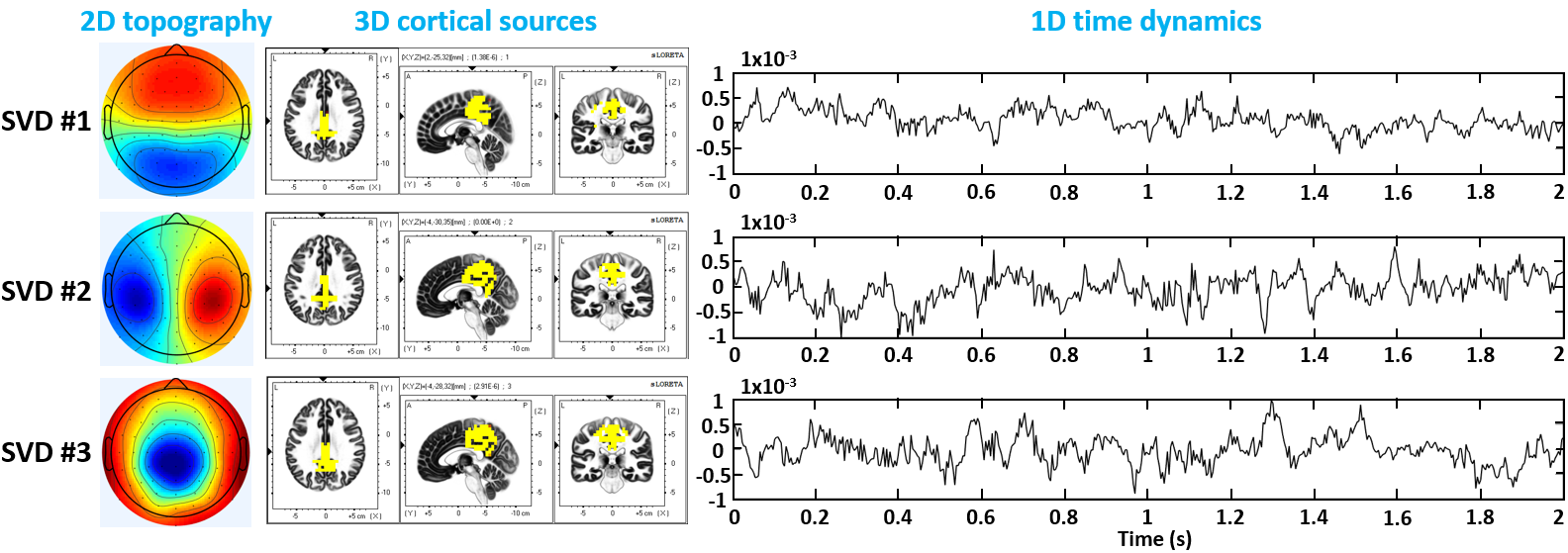


**Figure 4** Demonstration of the 2D electrical potential distribution (leftmost column), 3D cortical neural activity (or current density) sources (middle column), and an example section of 2-s time dynamics (rightmost column) of the first 3 SVD components or brain networks from one subject. Although they seem to share similar 3D cortical source locations, their 2D electrical potential distribution and temporal series are distinct (PCC<0.07 between each pair of the three components), suggesting three independent brain networks.

**Table 1.** Potential match between gSVD-derived EEG networks with fMRI-recognized networks

| **SVD brain networks** | **Brodmann Area** | **Different names of fMRI networks used in literatures** |
| --- | --- | --- |
| **#1** | 7, 23, 31 | posterior default mode [68-73] |
| **#2** | 23, 24, 31 | posterior default mode [68-73] |
| **#3** | 7, 23, 31 | posterior default mode [68-73] |
| **#4** | 39, 45, 47 | left working memory [73], central executive [68], left frontal-parietal [69, 71], left dorsal visual [70] |
| **#5** | 7, 39, 47 | right working memory [73], central executive [68], right frontal-parietal [69, 71], right dorsal visual [70] |
| **#6** | 6, 8, 32 | executive control [69, 70, 124], frontal attention [73], medial frontal network [69, 71] |
| **#7** | 1, 3, 40 | sensory-motor [69-73] |
| **#8** | 7, 18, 19, 40 | occipital pole visual [69], ventral visual [73], lateral visual [70] |
| **#9** | 10, 11 | anterior default mode [68, 70-73] |
| **#10** | 18, 19 | occipital [74], primary visual [75] |
| **#11** | 18, 19 | occipital [74], primary visual [75] |
